# Antiviral Protein–Protein Interaction Inhibitors

**DOI:** 10.1021/acs.jmedchem.3c01543

**Published:** 2024-02-23

**Authors:** Violeta Marković, Anna Szczepańska, Łukasz Berlicki

**Affiliations:** † Wrocław University of Science and Technology, Department of Bioorganic Chemistry, Wyb. Wyspiańskiego 27, 50-370 Wrocław, Poland; ‡ University of Kragujevac, Faculty of Science, Department of Chemistry, R. Domanovića 12, 34000 Kragujevac, Serbia

## Abstract

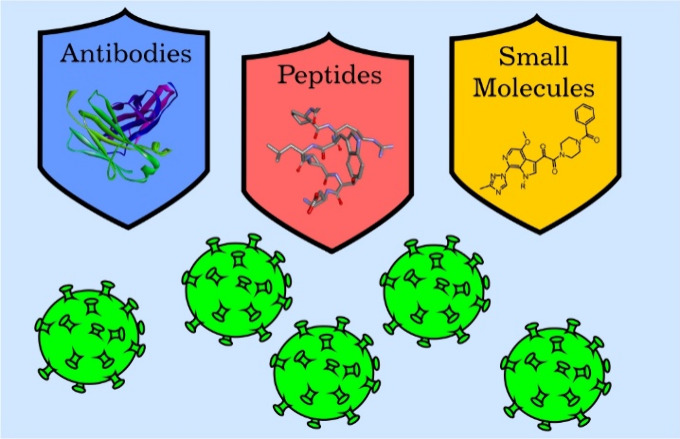

Continually repeating outbreaks of pathogenic viruses necessitate the construction of effective antiviral strategies. Therefore, the development of new specific antiviral drugs in a well-established and efficient manner is crucial. Taking into account the strong ability of viruses to change, therapies with diversified molecular targets must be sought. In addition to the widely explored viral enzyme inhibitor approach, inhibition of protein–protein interactions is a very valuable strategy. In this Perspective, protein–protein interaction inhibitors targeting HIV, SARS-CoV-2, HCV, Ebola, Dengue, and Chikungunya viruses are reviewed and discussed. Antibodies, peptides/peptidomimetics, and small molecules constitute three classes of compounds that have been explored, and each of them has some advantages and disadvantages for drug development.

## Significance

- Protein–protein interaction inhibitors are a highly important and emerging group of antiviral agents.
- Numerous successful examples of compounds targeting HIV, SARS-CoV-2, HCV, Ebola, Dengue, and Chikungunya viruses are discussed.
- Improving methodologies of protein–protein interaction inhibitor design and development make this group of molecules more attractive in comparison to widely explored enzyme inhibitors.

## Introduction

Viruses have coexisted with humans forever, and they cause epidemics of various geographical ranges and intensities in a regular fashion.^1^ Recently, these outbreaks have included severe acute respiratory syndrome (SARS) (2002), influenza A/H1N1 (2009), Ebola (2013), and SARS-CoV-2 (2019) viruses. Due to the high propensity of viruses to mutate, new viral pathogens can be expected to continuously threaten humans.^2^ Therefore, the development of antiviral strategies is and will remain highly important in the future.^3,4^ Taking into consideration the molecular mode of action, enzyme inhibitors are studied most intensively due to the importance of enzymes in the viral replication process as well as the well-established experimental and computational methodologies of enzyme inhibitor discovery.^5,6^ However, fast changes in viruses necessitate the application of various strategies; thus, other options, including protein–protein interaction inhibitors, are also of high importance.

Protein–protein interactions (PPIs) regulate a large number of processes in biological systems and thus are often targets for the treatment of various diseases.^7,8^ Although major efforts in this field are focused on the development of PPI inhibitors with anticancer activity, other applications of this strategy are also of high importance. The discovery of antiviral PPIs has already been significant, over a 20-year-long history, with the first drug (Enfuvirtide) approved by the FDA in 2003. The use of PPI inhibitors in the treatment of various viral infections has already been shown.

The most explored strategy is related to the construction of virus entry inhibitors and, thus, the interaction of human cell surface proteins with virus capsid proteins. It has already been shown to be effective and marketed for anti-HIV treatment, while drug candidates for HCV, SARS-CoV-2, and other viruses have been found. The HIV fusion process includes interactions of gp41 and gp120 of HIV and CD4, CCR5, and CXCR4 of humans. In the case of SARS-CoV-2, the interaction of the viral spike protein with human ACE2 is crucial for entry. HCV interacts with several surface human proteins, including SR-BI, CD81, CLDN1, and OCLN. Notably, other types of protein–protein interactions that can be targeted by antivirals; e.g., inhibitors of HIV protease or reverse transcriptase dimerization or oligomerization of integrase, were investigated.

Three major approaches for the development of antiviral protein–protein interaction inhibitors can be distinguished, namely, low-molecular-weight compounds,^9,10^ peptide-based inhibitors,^11,12^ and antibodies. Small molecules are usually challenging to develop because of the discrepancy between the large surface of the interaction between proteins and their size. In particular, small molecules can bind to one hot spot, unless other hot spots are nearby. However, there are some successful stories in this area, and two drugs, maraviroc, which binds to CCR5, and fostemsavir, which interacts with gp120, have been marketed. On the other hand, medium-sized compounds, including peptides and peptidomimetics, have been effectively explored and provide highly active drug candidates. Their size allows for reaching several spatially separated hot spots. In recent years, antiviral peptides have gained increasing interest as highly specific and effective potential therapeutics of natural or computational origin with diverse activities and minimal toxicity. Enfuvirtide, a 36-mer peptide, is the first marketed drug of this type. Finally, antibodies, in spite of their disadvantages, can also be used to develop inhibitors of protein–protein interactions, for example, ibalizumab, an anti-HIV drug binding to CD4.

In summary, various strategies for the construction of protein–protein interaction inhibitors of antiviral activity will be discussed in this perspective article. This concerns both the analysis of possible molecular targets and the possibility of the application of compounds derived from various classes.

## HIV Inhibitors

Acquired immunodeficiency syndrome (AIDS) develops from the infection and subsequent depletion of T lymphocytes caused by the human immunodeficiency viruses (HIV-1 and HIV-2).^13,14^ With more than 38 million people living with HIV, this global epidemic continues to present a significant global health problem, and the development of effective new treatment strategies and medicines remains a critically important challenge.^15^ The greater infectivity of the HIV-1 type, which is more readily transmitted, makes it predominant and responsible for the AIDS pandemic.^16^

The process of HIV-1 entering and infecting a target cell starts with the interaction between an HIV-1 envelope glycoprotein (HIV-1 Env), gp120, and a cellular receptor protein, CD4. Mutual attachment leads to conformational changes in gp120 that prompt its binding to a coreceptor on the cell surface (C–C chemokine receptor type 5, CCR5 or C-X-C chemokine receptor type 4, CXCR4). This binding triggers a conformational rearrangement of another HIV-1 envelope glycoprotein, gp41, causing an interaction with the host cell membrane and resulting in the fusion of the viral envelope with the cellular membrane.^17,18^ Consequently, HIV-1 invasion can be divided into four stages: (1) viral attachment to the host cell; (2) gp120-CD4 binding; (3) gp120-coreceptor binding; and (4) fusion between the virus and cell membrane. Therefore, gp120, CD4, coreceptors (CCR5 or CXCR4), and gp41 are all essential elements in the entry process and represent attractive drug targets.^19^ Antiviral agents that may disrupt any step of the presented process can prevent viral entry and are categorized as HIV-1 virus entry inhibitors. It is important to point out that entry inhibitors are typically used in combination with other antiretroviral drugs to form highly active antiretroviral therapy (HAART) regimens, predominantly for “heavily treatment experienced” (HTE) individuals with limited treatment options, usually due to extensive drug resistance. According to the steps of the viral entry process, entry inhibitors can be broadly classified into four categories: (1) preattachment inhibitors, blocking the first phase of viral attachment; (2) postattachment inhibitors, binding to CD4 receptors and inducing conformational changes in the CD4–gp120 complex; (3) CCR5 antagonists, binding to the coreceptor CCR5 and preventing gp120–coreceptor attachment; and (4) fusion inhibitors, interacting with gp41, preventing viral and host cell membranes from coming into close proximity and disabling membrane fusion. Four HIV-1 entry-inhibitor-based medicines have been approved by the U.S. Food and Drug Administration (FDA) (Table 1).

**Table 1:** FDA-Approved HIV-1 Entry Inhibitors

generic name	structure type	drug class
Ibalizumab	antibody	postattachment inhibitor
Enfuvirtide	peptide	fusion inhibitor
Fostemsavir	small molecule	preattachment inhibitor
Maraviroc	small molecule	CCR5 antagonist

### Antibody-Based Inhibitors

Antibody-based therapies possess several advantages over small molecule-based treatment in various aspects, such as a high degree of specificity for the target and safety.^20^ Treatment with a monoclonal antibody (mAb) brings several advantages for HIV therapy, including a unique mechanism of action, capacity to restore CD4 T-cell counts, prolonged time to resistance development, and a low potential for toxicities.

**Figure 1:**
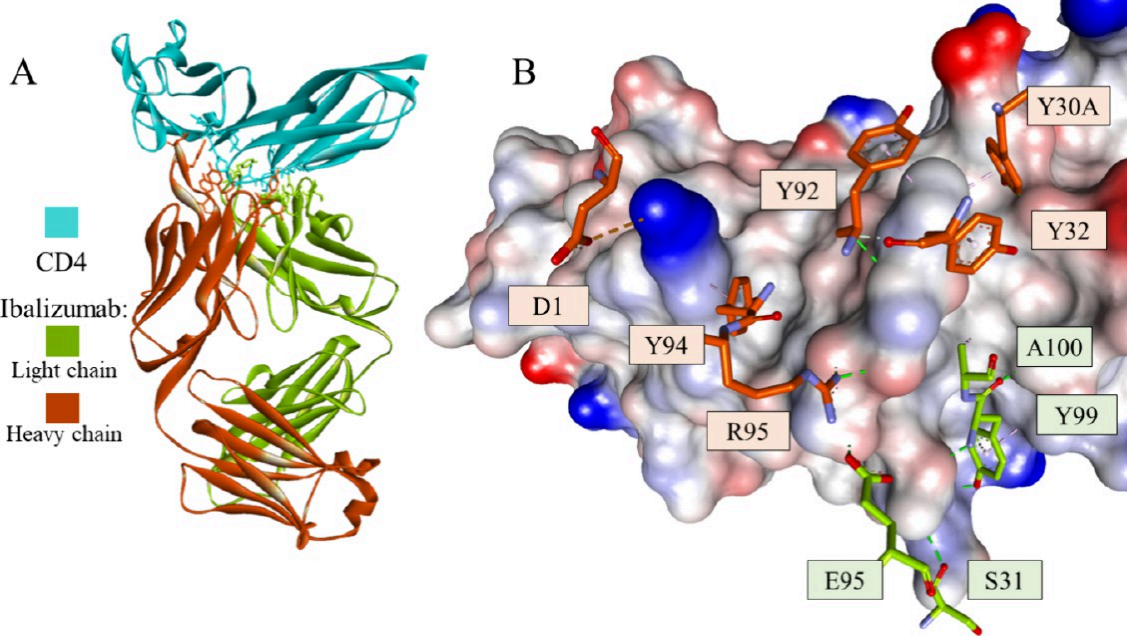
Crystal structure of the ibalizumab-CD4 complex (PDB id: 3O2D) (A) and a fragment of CD4 with selected interacting residues of the antibody (B). CD4 is shown as a cyan ribbon or as a solvent-accessible surface colored by interpolated charge (blue–positive, gray–neutral, red–negative). The antibody is shown as a green or orange ribbon for the light and heavy chains, respectively. Interacting residues are shown in stick representation, with carbon atoms colored the same as the parent chain. Intermolecular interactions are shown as dashed lines: green–hydrogen bonds, orange–charge-assisted hydrogen bonds, pink–hydrophobic interactions, and white–hydrogen bond donor/π interactions.

Ibalizumab (TNX-355) is a humanized IgG4 mAb that targets the CD4 receptor and blocks HIV-1 infection.^21^ A phase 3 trial demonstrated that ibalizumab is a safe and well-tolerated drug and can be used as a monotherapy, especially for multidrug-resistant HIV patients with limited treatment options.^22^ Researchers are currently developing a new formulation of ibalizumab for intramuscular injection use. Studies suggest that ibalizumab does not inhibit the process of binding of gp120 to CD4, which occurs in the domain 1 region. It appears to exert its antiviral effect by postbinding conformational effects that prevent CD4-bound gp120 from interacting with CCR5 or CXCR4 without interfering with major histocompatibility complex class II (MHC-II)-mediated immune function.^23^ This is consistent with the fact that ibalizumab is well tolerated when introduced to patients and has demonstrated promising anti-HIV-1 activity without causing immunosuppression. The crystal structure of the ibalizumab Fab fragment in complex with the two N-terminal domains of human CD4 was reported and confirmed binding of ibalizumab on the opposite side of both gp120 and MHC class II binding sites (Figure 1).^24^

**Figure 2:**
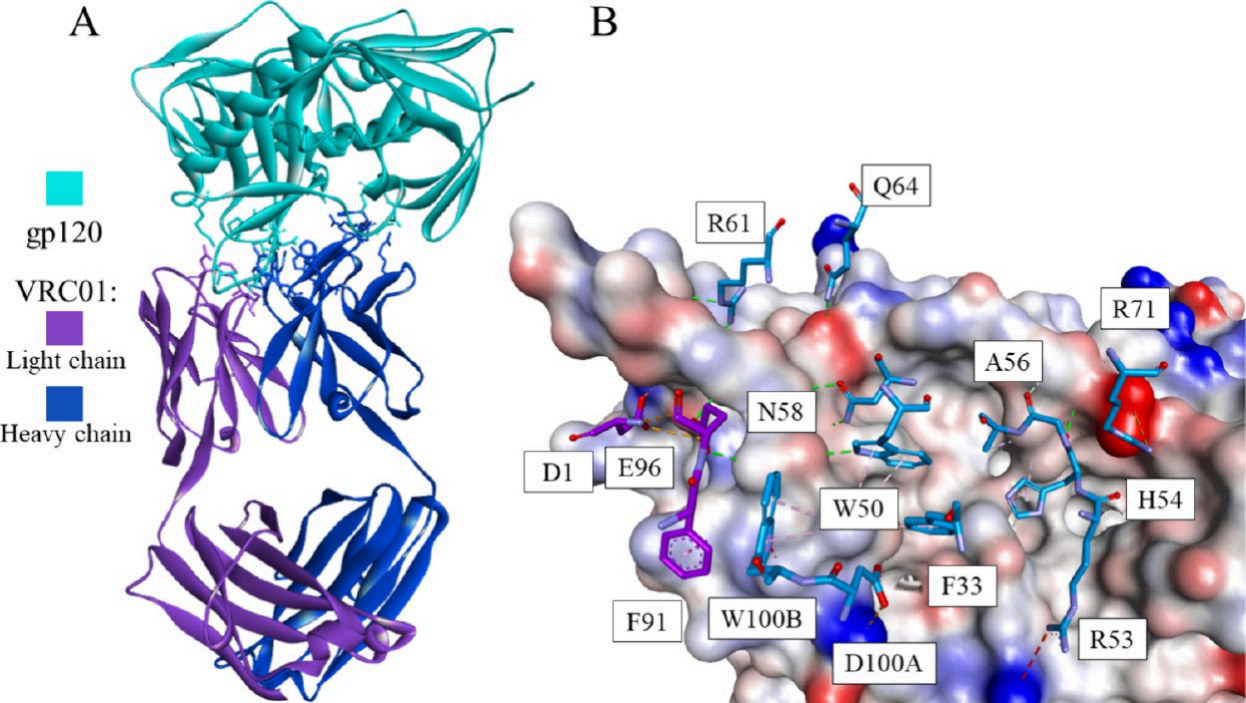
Crystal structure of the VRC01 antibody-gp120 complex (PDB id: 5CD5) (A) and a fragment of gp120 with selected interacting residues of the antibody (B). gp120 is shown as a cyan ribbon or as a solvent-accessible surface colored by interpolated charge (blue–positive, gray–neutral, red–negative). The antibody is shown as violet or dark blue ribbons for the light and heavy chains, respectively. Interacting residues are shown in stick representation with carbon atoms colored the same as the parent chain. Intermolecular interactions are shown as dashed lines in the same color as in Figure 1.

Broad neutralizing antibodies (bnAbs) are capable of neutralizing multiple HIV-1 viral strains by targeting conserved epitopes of viruses and have significantly contributed to HIV vaccine development.^25^ However, none of the FDA-approved therapeutic antibodies are used for HIV treatment. One of the bnAbs, VRC01, effectively suppressed plasma viremia below detectable concentrations in a phase I trial, but the emergence of VRC01-resistant HIV after exposure is the main concern.^26^ The crystal structure of VRC01 in complex with an HIV-1 gp120 core showed that the heavy chain of VRC01 interacts with gp120 in a manner similar to CD4 (Figure 2).^27^ The results of a phase IIa trial using the HIV Env-specific antibody 3BNC117 showed that the antibody exerts strong selective pressure on HIV-1 emerging from latent reservoirs.^28^ In comparison to VRC01 and other less potent antibodies, 3BNC117 exhibited higher efficiency, which could be justified by its increased potency and/or a longer half-life.^29,30^ Research on anti-HIV therapeutic antibodies is continuing, and many of them have successfully advanced to human clinical trials.^31^

Various antibodies targeting CCR5, such as HGS004, PRO140, and cenicriviroc, have shown promising antiviral effects and are now being evaluated in clinical trials.^32−34^ An anti-PDL1 antibody, BMS-936559, has been subjected to a phase I trial on HIV patients, showing that the immunologic check point inhibitor could enhance HIV-specific immunity in a subset of participants included in the trial.^35^

### Peptide-Based Inhibitors

Peptide-based inhibitors, compared to small molecules, have the potential to be more efficient and specific due to larger surface areas, better target surface recognition, and potentially lower toxicity.^36^ Nevertheless, there are serious disadvantages limiting the application of peptides, such as proteolytic instability, conformational flexibility, and poor cellular penetration.^37^

**Figure 3:**
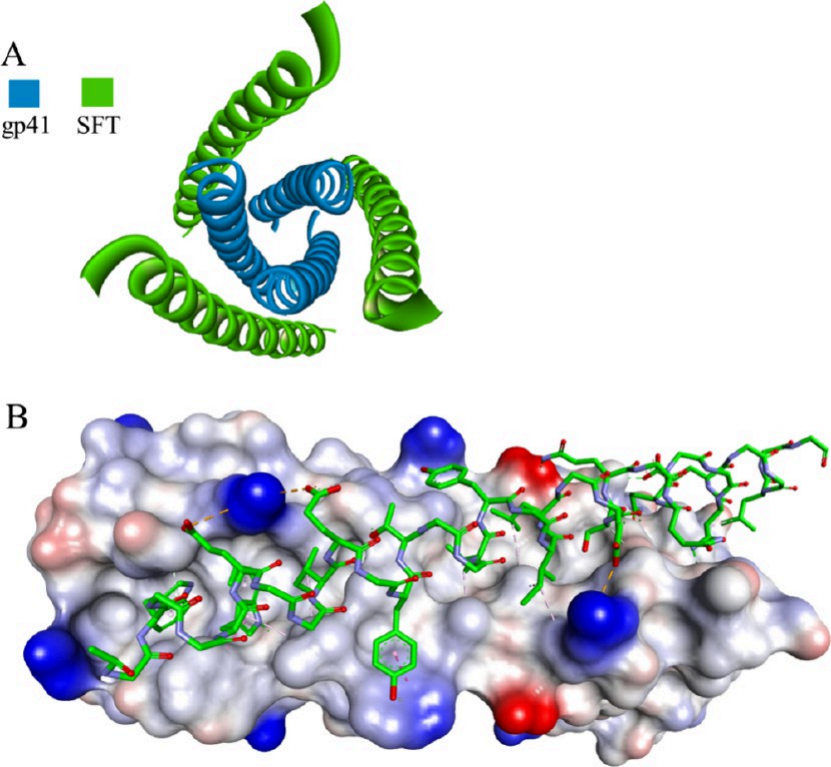
Crystal structure of the SFT-gp41 complex: top (A) and side view (B) (PDB id: 3VIE). gp41 is shown as a blue ribbon or as a solvent-accessible surface colored by interpolated charge (blue–positive, gray–neutral, red–negative). The SFT peptide is shown as a green ribbon or in stick representation colored according to atom types, and noninteracting side chains are hidden for clarity. Intermolecular interactions are shown as dashed lines in the same color as in Figure 1.

Several synthetic peptides derived from the NHR (N-terminal heptad repeat regions) and CHR (C-terminal heptad repeat regions) of gp41 can proficiently inhibit HIV-1 infection by competitively binding to the exposed NHR or CHR in the gp41 prehairpin state, therefore blocking six-helix bundle (6-HB) formation in a dominant-negative manner.^38,39^ Several pairs of protease-resistant N- and C-peptides from gp41, including N36 and C34, which were later found to form the stable fusogenic core, were identified.^40^ The peptide C34 is more potent in inhibiting HIV-1 fusion than both SJ-2176^41^ and T-20.^42^ It was proposed that C-peptide (e.g., C34) may be involved in blocking the formation of the fusion-active core of gp41 and inhibiting the fusion between the viral and target cell membranes.^43^ Enfuvirtide (T20/Fuzeon), a native CHR-derived peptide with 36 amino acids, was approved for clinical use in 2003 as a first member of a new class of anti-HIV drugs - HIV fusion inhibitors.^44^ However, it was determined that this peptide drug easily induces resistance in both clinical settings and laboratory studies.^45^ As a response to the drug resistance problem, many efforts have been undertaken to design and develop new anti-HIV agents including fusion inhibitors with improved stability and potency. The problem of fibrillation as a common issue in the development of therapeutic peptides has been addressed recently, and cucurbit[7]urils (CB[7]), a group of water-soluble macrocycles, was reported. These compounds are capable of modulating fibrillation behavior of the HIV fusion inhibitor enfuvirtide by specifically binding to the C-terminal Phe residue.^46^ The second-generation peptide, the fusion inhibitor T1249, exhibited enhanced antiviral potency, but its clinical development was discontinued owing to the drug formulation problem.^47^ Sifuvirtide (SFT) is a third-generation peptide-based HIV-1 fusion inhibitor approved for phase III clinical trials in China.^48^ It is an electrostatically constrained α-helical peptide fusion inhibitor showing potent anti-HIV activity, good safety, and pharmacokinetic profiles. The crystal structure of SFT in complex with its target NHR sequence was solved by using the peptide N36 as a surrogate (Figure 3).^49^ This reveals that SFT adopts a fully helical conformation stabilized by multiple engineered salt bridges that involve, in addition to the residues at the *i* and *i* + 4 positions, other charged residues contributing to stabilization.

**Figure 4:**
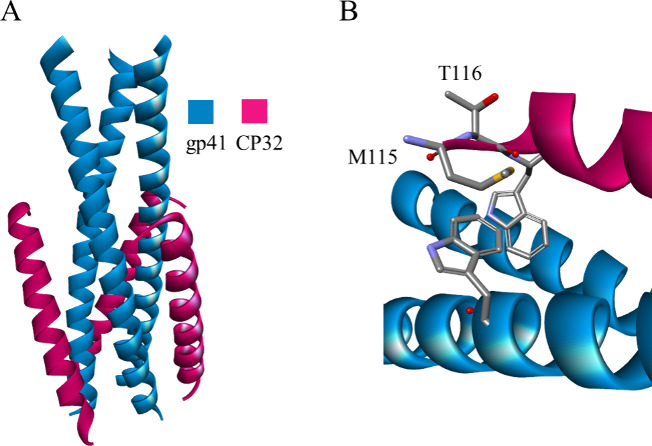
Crystal structure of the CP32 peptide-gp41 complex (A) and enlargement of the hook region (B) (PDB id: 3VTP). Gp41 and CP32 are shown as blue and red ribbons, respectively. The hook region is shown in stick representation colored according to atom types.

The crystal structure of another CHR-based peptide, CP32, with high efficiency against both enfuvirtide- and C34-resistant HIV-1 strains,^50^ showed that the residues Met115 and Thr116 preceding the pocket-binding domain (PBD) of the peptide form a hook-like structure, named the M-T hook (Figure 4).^51^

The structure of this molecule was modified with 11 of 32 residues mutated, giving peptide CP32M, which exhibited significant anti-HIV activity against enfuvirtide-resistant viruses.^52^ Positively or negatively charged residues were introduced to promote the formation of ion pairs (salt bridges) or to increase the hydrophilicity of the peptide. The findings related to the structure of CP32 also led to the introduction of the M-T hook into the Sifuvirtide structure, and the resulting peptide (MT-SFT) showed enhanced binding affinity and anti-HIV activity compared to Sifuvirtide, as well as an increased genetic barrier to the development of drug resistance.^53^ This strategy was also adopted in designing other short-peptide fusion inhibitors, such as a 24-residue peptide named MT-SC22EK by adding two hook residues to the N-terminus of the poorly active short-peptide SC22EK, which did show high activity against diverse HIV-1 variants.^54^ The crystal structures of these two peptides led to the development of a highly potent short-peptide inhibitor named HP23,^55^ whose structure was improved by the substitution of the methionine residue in the M-T hook structure with leucine to avoid potential oxidation problems, thus resulting in the inhibitor HP23L.^56^ Additionally, different lipids were introduced into the C-terminus of this inhibitor through a PEG linker, and one of these derivatives, termed LP-11, demonstrated strong anti-HIV activity. The M-T hook strategy has also been employed to design a fusion inhibitor, a 23-mer helical peptide named 2P23 that was effective against both HIV-1 and HIV-2 isolates.^57^ A lipopeptide derivative, LP-19, with improved binding stability and antiviral activity, was subsequently obtained by adding a fatty acid group to the C-terminus of 2P23.^58^ Moreover, it was shown that LP-19 exhibits a broad spectrum anti-HIV activity, and high drug resistance barrier compared to C34.^59^

Sulfonyl-γ-AApeptides that were designed to mimic MT-SC22EK, and showed comparable activity to this peptide, exhibited exceptional resistance to proteolysis, favorable PAMPA (parallel artificial membrane permeability assay) permeability, as well as promising oral bioavailability.^60^ Further investigations indicated that these sulfonyl-γ-AApeptides act by mimicking the CHR of gp41 and tightly bind NHR, resulting in inhibition of the 6-HB structure formation.

**Figure 5:**
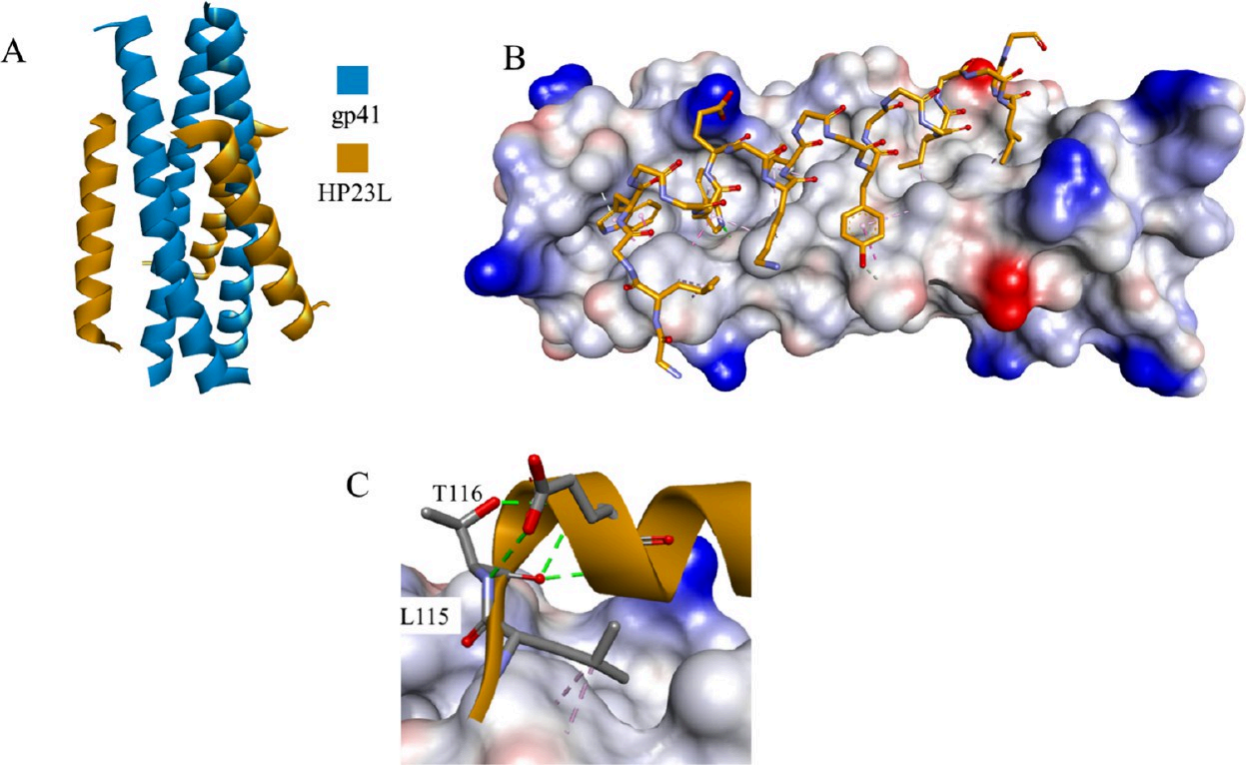
Crystal structure of the HP23L-gp41 complex (PDB id 5YB3) (A). Intermolecular interactions stabilizing the complex (B) and the structure of the L-T hook (C). gp41 is shown as a blue ribbon or as a solvent-accessible surface colored by interpolated charge (blue–positive, gray–neutral, red–negative). The HP23L peptide is shown as an orange ribbon or in stick representation colored according to atom types, and noninteracting side chains are hidden for clarity. Intermolecular interactions are shown as dashed lines in the same color as in Figure 1.

The crystal structures of HP23L and LP-11 bound to a target-mimic NHR peptide with the wild-type sequence or resistant mutations were determined (Figure 5A).^61^ The interhelical hydrogen bonds and salt bridges between HP23L and gp41 critically determined the binding of the inhibitors, and rich hydrophobic interactions played fundamental roles in stabilizing the whole structure (Figure 5B). Leu-115 and Thr-116 adopted a hooklike (designated L-T hook) structure, with Leu-115 forming numerous hydrophobic interactions with multiple pocket-forming residues, strengthening the stability of the L-T hook structure and the binding of inhibitors. Additionally, hydrogen bonds in the L-T hook region increased the binding stability of the hook and pocket region (Figure 5C).

**Figure 6:**
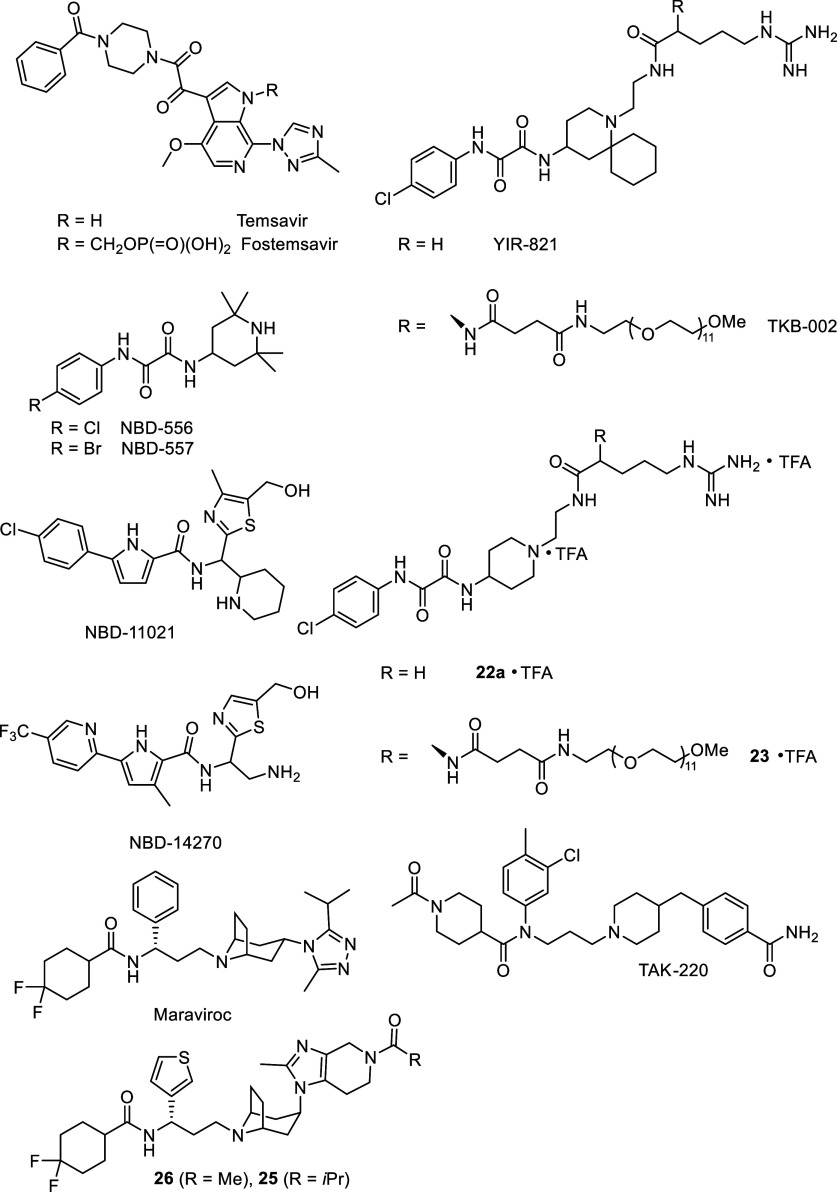
Structures of small molecule HIV entry inhibitors.

A multitarget-directed ligand strategy was used to design entry inhibitors that combine the pharmacological activities of a peptide fusion inhibitor disrupting HIV-1 gp41 glycoprotein hexameric coiled-coil assembly and a small-molecule CCR5 antagonist.^62^ Among these, dual-target 23-residue peptides SP12T and SP12L showed significantly increased inhibitory activities against HIV-1 replication in comparison to enfuvirtide. The same research group designed and engineered a chimeric peptide-based bifunctional HIV-1 entry inhibitor, AP3P4E, by incorporating the TAK-220 structure (Figure 6) to an artificially designed gp41-binding peptide, resulting in improved antiviral capabilities compared to parent components, as well as HIV-1 fusion inhibitor, enfuvirtide.

### Small-Molecule Inhibitors

Small-molecule HIV-1 entry inhibitors targeting early events in the virus life cycle represent an important class of valuable drugs. Their application has certain advantages in terms of lower cost, longer half-life *in vivo*, and oral availability, making them more convenient to use than, for example, peptide-based therapeutics. The first in action, preattachment inhibitors, are responsible for gp120–CD4 interaction inhibition, thus preventing the first stage of viral attachment to the target cell. One of the most effective entry inhibitor classes targeting the HIV-1 Env gp120 subunit was developed by Bristol-Myers Squibb and includes a group of compounds sharing a piperazine core motif (Figure 6).^63^

**Figure 7:**
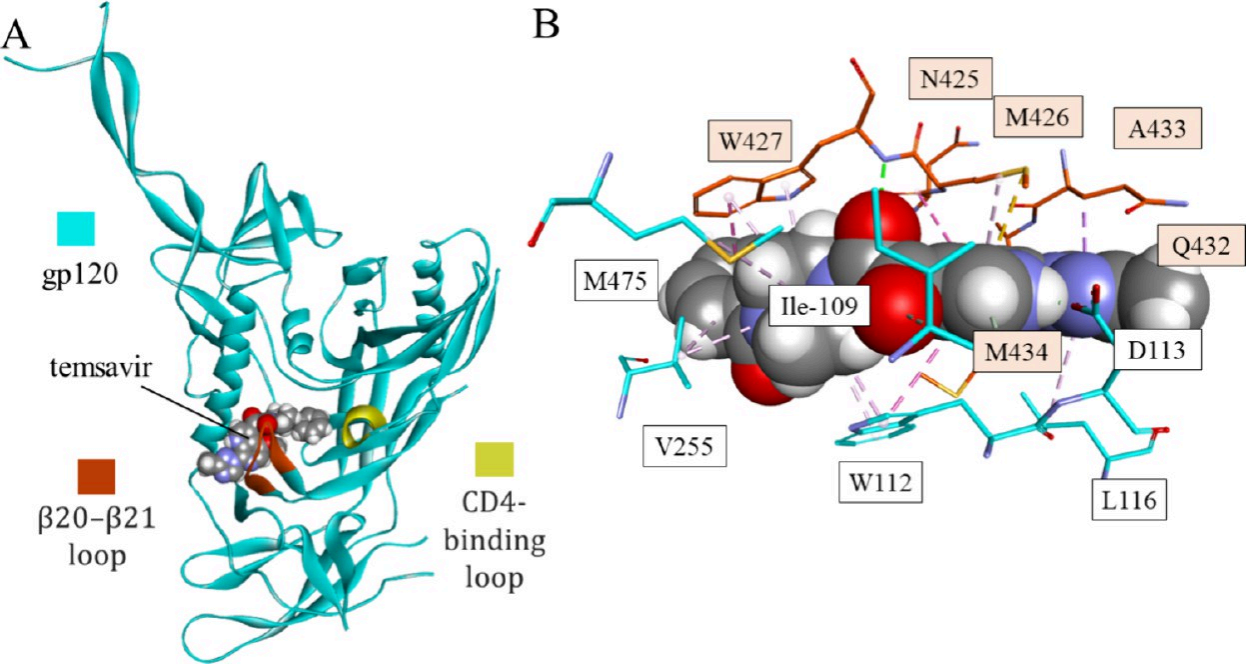
Mode of binding of temsavir to gp120: the temsavir-gp120 complex (A) and closeup of the binding cavity of gp120 with interactions between temsavir and the target (PDB id 5U7O). gp120 is shown as a cyan ribbon with β20-β12 and CD4-binding loops colored red and yellow, respectively. Interacting residues (B) are shown in stick representation colored according to atom type and carbon atom color that matches the parent chain color. Temsavir is shown as space-filling balls. Intermolecular interactions are shown as dashed lines in the same color as in Figure 1.

From this class of inhibitors, temsavir (BMS-626529) showed potency against many of the major subtypes of HIV-1, but due to its low solubility, it showed poor bioavailability. Fostemsavir (BMS-663068), a phosphooxymethyl prodrug of temsavir designed to address dissolution- and solubility-limited absorption issues, binds to gp120, disabling the conformational rearrangements normally activated by CD4 binding and ultimately resulting in fusion of the virus to the target cell.^64^ Seventeen clinical studies on the pharmacodynamics, pharmacokinetics, drug–drug interactions, safety, and bioavailability of fostemsavir and temsavir have been completed, with five ongoing clinical studies, including one phase IV clinical trial.^65^ Fostemsavir has performed well in clinical trials and was approved for use by the FDA in 2020. However, studies indicated suboptimal solubility after cleavage of the prodrug and scope concerns for specific subtypes of HIV-1. For this reason, fostemsavir is only suggested for treatment-experienced patients with limited therapeutic options and developed resistance to multiple existing antiretroviral drugs. The X-ray structure of the cocrystal complex of gp120 with temsavir revealed an induced binding pocket under the β20–21 loop (Figure 7), distinct from the docking studies that place this type of compound in the Phe43 cavity.^66^

CD4 mimetics (CD4m) are a class of compounds designed to competitively bind to HIV gp120 protein, effectively blocking its interaction with the CD4 receptor and preventing viral entry. Keeping in mind that the Phe43 cavity of the HIV-1 gp120 envelope glycoprotein plays a crucial role in the interaction with the CD4 receptor during HIV entry, there is continued interest in developing CD4 mimetics that specifically target the Phe43 pocket to inhibit viral entry. CD4m compounds were initially identified with the discovery of NBD-556 and NBD-557 (Figure 6), two small molecules composed of an aromatic ring, an oxalamide linker, and a piperidine moiety.^67^ These molecules inhibit HIV-1 entry into cells expressing CD4 and a coreceptor.^68^ The cocrystal structure of NBD-556 bound to the extended gp120 core demonstrated that the oxalamide moiety formed two hydrogen bonds with backbone carbonyls of residues on opposite sides of the Phe43 cavity.^69^ Further modification of the NBD-556 structure led to new CD4 mimics, such as YIR-821 (Figure 6), containing a cyclohexane group in a spiro attachment to a piperidine ring and a guanidino group on the piperidine nitrogen atom.^70^ These molecules were designed to establish hydrophobic and electrostatic interactions with Val430 and Asp368 located in the entrance of the Phe43 cavity of gp120. These interactions were confirmed by the molecular modeling of YIR-821 docked into gp120. This compound exhibited remarkable synergistic anti-HIV activity when coadministered with the neutralizing antibody KD-247, thus representing a promising lead, especially for use with neutralizing antibodies in combination therapy. To improve the low water solubility of the NBD-556 molecule that might correlate with relatively strong cytotoxicity, the hydrophobic aromatic ring was replaced with various pyridine-type moieties (Figure 6).^71^ Some of these derivatives showed high anti-HIV activity, indicating that a halogen substituent on the carbon closest to the nitrogen atom in the pyridine ring can conserve anti-HIV activity. In addition, the CD4ms that have a cyclohexyl group instead of a tetramethyl group on the piperidine ring also showed high anti-HIV activity and no significant cytotoxicity. Recently, TKB-002, a hybrid CD4m containing a polyethylene glycol unit attached through an uncleavable linker, was developed and showed high anti-HIV activity and low cytotoxicity (Figure 6).^72^ This hybrid compound showed a more effective pharmacokinetic profile when tested in a rhesus macaque than did the parent compound, YIR-821. In addition, novel compounds were designed and synthesized, in which the phenyl group of YIR-821 and TKB-002 was replaced by a halopyridyl group with the halogen atoms and the nitrogen atom on the pyridine ring at different positions.^73^ Additionally, compounds without cyclohexane groups on the piperidine ring were prepared to increase the water solubility and decrease the hydrophobicity and cytotoxicity. Two of these compounds, **22a** and **23**, maintained promising anti-HIV activity but below that of YIR-821 (Figure 6).

The development of NBD-556-based entry inhibitors has been compromised after the discovery of its properties as CD4 agonist and its ability to promote HIV-1 infectivity in CD4-CCR5+ cells.^74^ A successful structure-based modification of the oxalamide midregion was achieved, and a CD4-antagonist named NBD-11021 was obtained (Figure 6, IC_50_ against Env-pseudotyped HIV-1 as low as 270 nM).^75^

**Figure 8:**
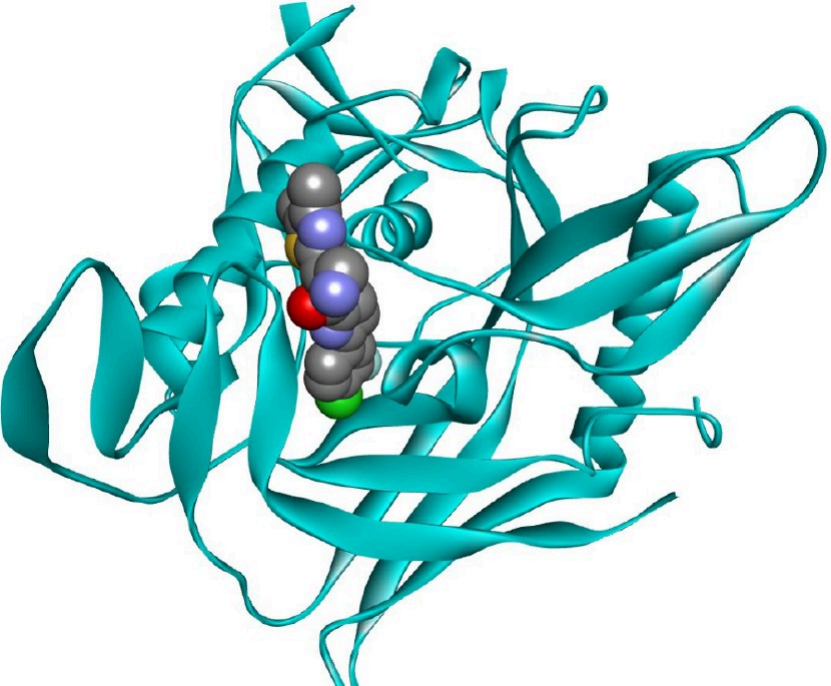
NBD-14010/CD120 complex, CD120 protein is shown as a cyan ribbon, and the inhibitor is shown as space-filling balls colored according to atom type.

The X-ray structure of NBD-11021 prompted further modification of the molecules in which the piperazine ring was replaced with an amine, giving NBD-14010 (Figure 8). The X-ray structure of NBD-14010 implied the modification of the thiazole ring substituents to obtain stronger interactions with the target protein.^76^ This led to the development of the hypothesis of the CH_2_OH “positional switch” that resulted in more potent antivirals, such as NBD-14136 (IC_50_ = 0.27 μM), NBD-14168 (IC_50_ = 0.28 μM), and NBD-14189 (IC_50_ = 0.089 μM).^77^ It was confirmed by X-ray analysis that the CH_2_OH group at position 5 in the thiazole ring showed no interactions, while switching of the CH_2_OH to position 4 in the thiazole ring, provides H bonding with Met426 and Gly431 (NBD-14189).^78^

To overcome the existing shortcomings of NBD-14189, such as high cytotoxicity and relatively poor aqueous solubility, the phenyl ring was replaced with a pyridine moiety.^79^ One of the new analogues, NBD-14270 (Figure 6), showed a noticeable enhancement in cytotoxicity, with 58-fold improvements in the selectivity index (SI) value as well as improvements in aqueous solubility compared to NBD-11021. A recent study that explored the replacement of the thiazole ring in NBD-14270 with two other positional thiazole isomers, revealed that the original scaffold provided the best HIV-1 inhibitors with a higher potency and better SI.^80^

The CD4m small molecule inhibitors BNM-III-170 and BNM-IV-147 were also developed to bind the Phe43 pocket but also to prevent infection of cells lacking CD4.^81^ Using single-particle cryo-EM, it was found that this inhibitor binds to the native-like BG505 Env trimer, resulting in its opening and structural rearrangements similar to those provoked by the CD4 host receptor.^82^

A carbamoyl derivative, named TAK-220 (Figure 6), showed good metabolic stability, potent binding affinity to CCR5 (IC_50_ = 3.5 nM), and excellent inhibition of membrane fusion (IC_50_ = 0.42 nM).^83^ For these reasons, it was further selected for Phase I clinical trials as a promising candidate for the treatment of HIV-1 infection.^84^

**Figure 9:**
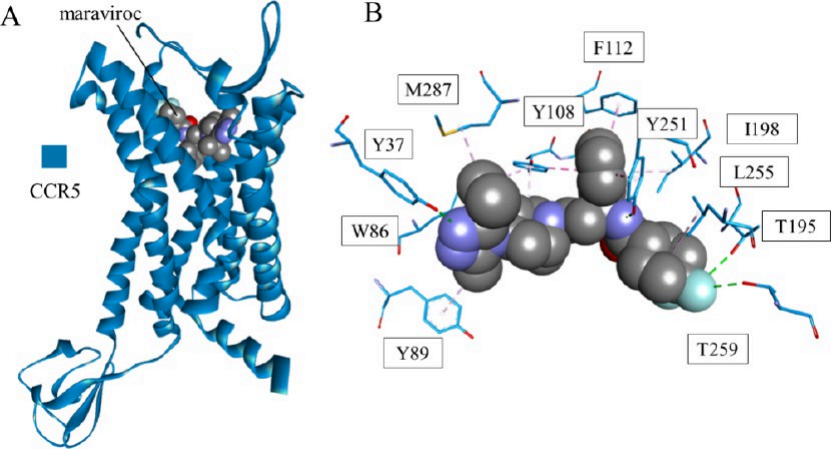
Crystal structure of maraviroc bound to CCR5 (PDB id: 4MBS) (A) and closeup of the interaction site of the drug (B). CCR5 is shown as a blue ribbon, and maraviroc is shown as space-filling balls colored according to the atom type. Interacting residues (B) are shown in stick representation colored according to the atom type, and the carbon atom color matches the parent chain color. Interactions are shown as dashed lines with the same color scheme as in Figure 1.

Maraviroc (Figure 6), a CCR5 antagonist, is a selective imidazopyridine molecule that received FDA approval for clinical use in 2007^85^ and is currently being used to treat patients with resistance to multiple HIV drugs but has also been recently approved for first-line treatment regimens.^86^ This compound displayed good antiretroviral activity, without blocking potassium channels, and good absorption.^87^ It is also characterized by a good pharmacokinetic profile, relatively low protein binding, and high bioavailability.^88^ The structural details of the maraviroc-CCR5 complex have been determined through X-ray crystallography, providing insights into the mechanism of allosteric inhibition of chemokine signaling and viral entry (Figure 9).^89^ Vicriviroc is another CCR5 inhibitor that has entered phase III clinical trials.^90^ However, due to the inferior results of this compound compared to other drugs, clinical studies in treatment-naive patients have been stopped, but the development of Vicriviroc for treatment-experienced patients continues.

Recently, the structure of the CCR5-maraviroc complex (Figure 9) was used for the design and synthesis of novel tropane derivatives.^91^ Two of them, compounds **26** and **25** (Figure 6), showed activity comparable to maraviroc, without significant cytochrome P450 (CYP450) inhibition. Furthermore, docking analysis of these compounds showed a new binding mode with CCR5 in comparison to maraviroc-CCR5, which implies that this could be a strategy to overcome the problem of drug resistance derived from maraviroc.

Broadly neutralizing antibodies (bnAbs) of HIV-1, such as VRC01, possess exceptional potency against variant strains of HIV-1.^92^ An *in silico* study on natural product-derived compounds that mimic VRC01 has delivered molecules that could serve as templates for the design of next-generation HIV-1 inhibitors, giving novel insights into the binding mechanisms using molecular dynamics simulations.^93^

## SARS-CoV INHIBITORS

**Figure 10:**
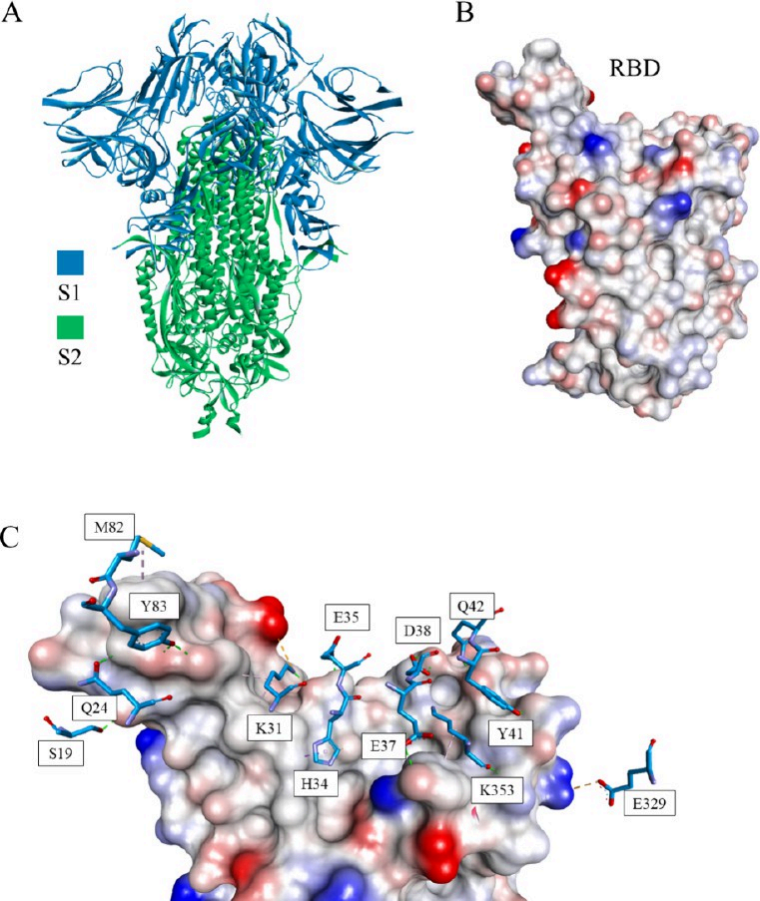
Crystal structure of the S protein (PDB id 6VXX) (A) and its receptor-binding domain (RBD) (B). S protein is shown in ribbon representation colored in blue and green for S1 and S2 chains, respectively. (C) The interactions between RBD and the S-protein have been shown with S1 represented as sticks (PDB ID: 6VW1).^99^ RBD is presented as a solvent-accessible surface colored by interpolated charge: red–negative, blue–positive, gray–neutral.

Coronaviruses are a group of related enveloped, single-stranded, positively sensed RNA viruses. Although some coronaviruses can cause the common cold, with mild symptoms, others are highly pathogenic and have led to several outbreaks in recent years.^94−96^ Severe acute respiratory syndrome coronavirus 2 (SARS-CoV-2) is the pathogen responsible for COVID-19, which was declared a pandemic and a global emergency shortly after it began in late 2019. Therapeutics currently used for COVID-19 treatment include convalescent plasma, neutralizing antibodies, and repurposed drugs. Coronavirus entry inhibitors play an important role in the treatment of coronavirus diseases alone or in combination with other drugs. They are designed to block various processes of viral entry, including receptor binding, proteolytic activation of spike protein, or virus–cell membrane fusion. The SARS-CoV-2 entry pathways are well understood, in part due to the close similarity of SARS-CoV-2 to SARS-CoV, the causative agent for a global outbreak in 2002–2003.^97^ SARS-CoV-2 Spike (S protein), a homotrimeric protein found on the surface of the viral membrane, mediates the main entry steps, including receptor binding and membrane fusion.^98^ Each monomer consists of two noncovalently associated subunits, S1, which is responsible for binding the receptor, and S2, which mediates membrane fusion (Figure 10).

The S1 subunit consists of an N-terminal domain (NTD) and a receptor binding domain (RBD), which contains a subdomain called the receptor binding motif (RBM).^100^ The first step in viral entry, and an attractive therapeutic target, is the interaction of S1 RBD and angiotensin converting enzyme-2 (ACE-2) of the host cell.^101^ The next step is mediated by the S2 subunit and involves fusion with the cell membrane, an entry point that can also be selectively targeted. The S2 subunit is located at the C-terminus and is built from a fusion peptide (FP), two α-helical heptad repeats (HR1 and HR2), a loop region, a transmembrane (TM) domain, and a cytoplasmic tail (CT). While the S1–S2 boundary is cleaved by furin in the virus-producing cell, the S2′ site cleavage requires target cell proteases, and serine 2 transmembrane protease (TMPRSS2) and cathepsin L are the two main proteases involved in the activation of the S protein. After the formation of the S-ACE2 complex, four pairs of disulfide bonds stabilize the RBD structure, while the RBM forms a concave outer surface to accommodate the N-terminal helix of ACE2. Additionally, the complex is further stabilized through 13 hydrogen bonds, two salt bridges (between K417 of the RBD and D30 of ACE2), and several hydrophobic interactions (between F486 of the RBD and L79, M82, and Y83 of ACE2).^102,103^ Among these, salt bridge interactions between K417 of the SARS-CoV-2 S protein and D30 of ACE2 are not present in SARS-CoV. To date, none of the examined SARS-CoV-2 entry inhibitors have been approved by the FDA for treatment. However, to help strengthen public health protection against the spread of COVID-19 infection, the FDA has issued an emergency use authorization (EUA) for a few diverse agents with different mechanisms of action.^104^

### Antibody-Based Inhibitors

The role of monoclonal antibodies (mAbs) in the inhibition of SARS-CoV-2 is to prevent viral attachment mostly by binding to a nonoverlapping epitope on the surface spike protein RBD with high affinity, thus preventing the virus from binding to the human ACE2 (hACE2) receptor.^105^ However, the application of mAbs has been limited due to the development of SARS-CoV-2 variants resistant to existing treatments.^106^ Although the majority of SARS-CoV-neutralizing human mAbs (hmAbs) specifically bind to the RBD of the S protein,^107,108^ there are hmAbs that do not use the same mechanism of inhibition as RBD-specific hmAbs and can bind to, for example, the S2 domain and neutralize the virus in the postbinding step of viral entry.^109^ This finding indicates that a mixture of antibodies recognizing distinct regions and targeting more than one step in viral entry is expected to be more efficient in neutralizing the virus and conquering the generation of escape mutants.

**Figure 11:**
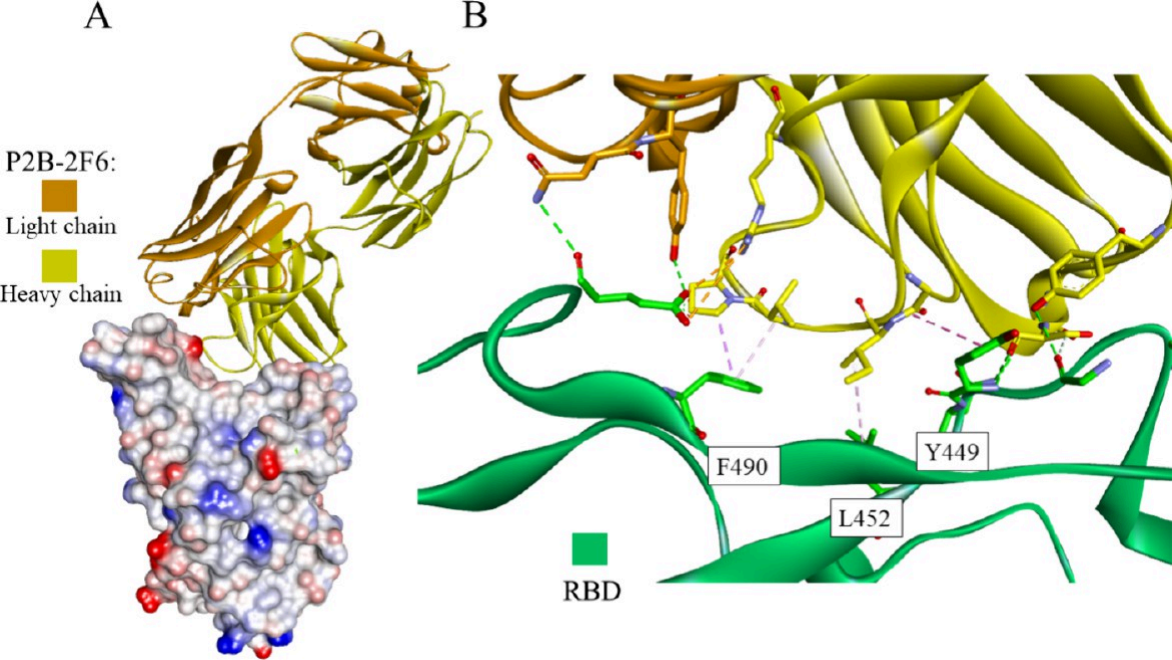
Crystal structure of the P2B-2F6 antibody - RBD of the S protein complex (PDB id: 8DCC): the whole structure (A) and closeup showing intermolecular interactions (B). The antibody shown in the ribbon representation is colored orange and yellow for light and heavy chains, respectively. The RBD is presented as a solvent-accessible surface colored by interpolated charge: red–negative, blue–positive, gray–neutral (A) or green ribbon (B). Interacting residues are shown as sticks colored by atom type, and the colors of the carbon atoms match that of the ribbon of the same chain. Interactions are shown as dashed lines with the same color scheme shown in Figure 1.

The high binding affinity of the antibody named P2B-2F6 to the RBD of SARS-CoV-2 (*K*_d_ = 5.14 nM) was found to be similar to that between ACE2 and the RBD (*K*_d_ = 4.70 nM), indicating ACE2 receptor engagement, which was also supported by the strong P2B-2F6 competition with ACE2.^110^ The crystal structure of P2B-2F6 in complex with the SARS-CoV-2 RBD indicates that P2B-2F6 attachment uses hydrophobic interactions around RBD residues Y449, L452, and F490 and hydrophilic interactions at the interface (Figure 11).

**Figure 12:**
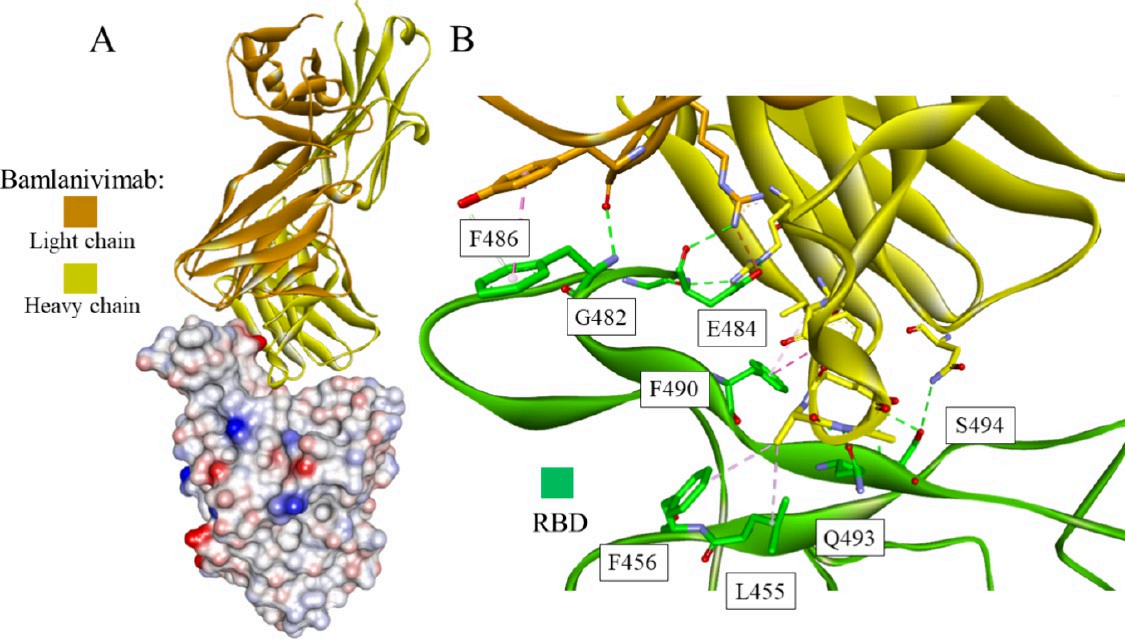
Crystal structure of bamlanivimab-RBD of the S protein complex (PDB id: 7KMG): the whole structure (A) and closeup showing intermolecular interactions (B). The antibody shown in ribbon representation is colored in orange and yellow for light and heavy chains, respectively. The RBD is presented as a solvent-accessible surface colored by interpolated charge: red–negative, blue–positive, gray–neutral (A) or as a green ribbon (B). Interacting residues are shown as sticks colored by atom type, and the color of the carbon atoms matches that of the ribbon of the same chain. Interactions are shown as dashed lines with the same color scheme shown in Figure 1.

Bamlanivimab (LY-CoV555 or LY3819253) is a potent neutralizing monoclonal antibody that targets the RBD of the SARS-CoV-2 S protein and has been shown to potently neutralize SARS-CoV-2.^111^ Structural analysis using X-ray crystallography and cryoelectron microscopy (cryo-EM) indicated that LY-CoV555 binds to an epitope overlapping the ACE2 binding site, and binding to the S protein RBD was observed in both up and down conformations (Figure 12). Bamlanivimab received EUA by the FDA at the end of 2020 for clinical use in nonhospitalized patients with mild to moderate conditions, but this decision was revoked after six months due to the emergence of SARS-CoV-2 variants resistant to bamlanivimab monotherapy.^112^ However, it is still used in combination with etesevimab, another monoclonal antibody.^113^

Bebtelovimab (LY-CoV1404) is a neutralizing monoclonal antibody targeting the S protein RBD of the SARS-CoV-2 virus and demonstrates broad neutralizing activity against all SARS-CoV-2 variants of concern (VOCs).^114^ Binding kinetic studies revealed that Bebtelovimab shows high affinity toward the S protein of D614G (*K*_d_ values between 790 pM and 4 nM). Both potent activity and structural analysis of binding of LY-CoV1404 to the RBD indicate that this antibody binds uniquely to an epitope with a low frequency of mutations. Bebtelovimab is equally effective at viral neutralization against all tested variants and several times more potent in viral neutralization assays than the antibody named VIR-7831, which also binds to an epitope of the SARS-CoV-2 S protein distinct from current VOCs and has clinical efficacy at a 500 mg dose.^115^

### Peptide-Based Inhibitors

Although highly effective experimental and computational methodologies for the development of peptide-based protein binders are available, the discovery of S protein–human ACE2 interaction inhibitors was found to be nontrivial.^116^

**Figure 13:**
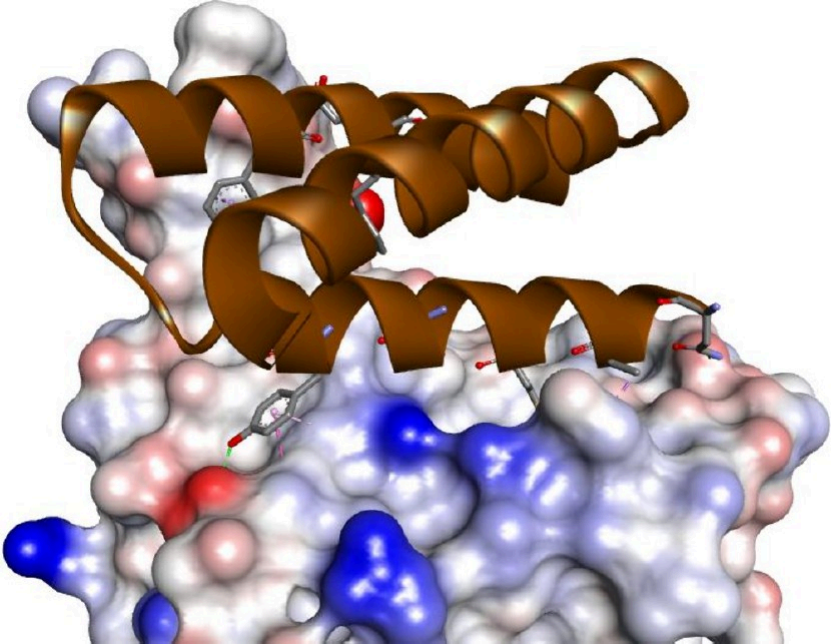
Cryo-EM structure of the small protein LCB3 bound to the RBD of the S protein (PDB id: 7JZM). LBC3 protein is shown as a brown ribbon with interacting residues represented as sticks colored according to the atom type. The RBD of the S protein is shown as a solvent-accessible surface that is colored according to the interpolated charge (red–negative, blue–positive, gray–neutral). Interactions are shown as dashed lines with the color scheme shown in Figure 1.

Highly active small proteins (56- and 64-amino acid long sequences) incorporating three helices were designed de novo using computational methodology.^117^ Two approaches were applied: (a) incorporation of the helical fragment of ACE2 into the binder structure and (b) building new proteins from scratch. A high number of computationally designed molecules were initially screened to observe their interaction with fluorescently labeled RBD displayed on the surface of yeast cells. The second optimization step relied on the production of saturation mutagenesis libraries and checking of the enrichment after fluorescence-activated cell sorting (FACS). One of the obtained proteins, LCB3, exhibited high conformational stability (*T*_m_ > 95 °C) combined with RBD binding *K*_d_ values below 1 nM and live virus neutralization EC_50_ values below 50 pM. The cryo-EM structure of the LCB3-S protein complex revealed numerous intermolecular interactions mediated mainly by two or three helices of LCB3 (Figure 13). Notably, its N-terminal helix positioning is similar to that of helix 1 of ACE2.

The discovery of shorter peptides that could inhibit ACE2–S protein interactions was difficult. Molecules that bind to RBD with *K*_d_ values in the nanomolar range up to *K*_d_ = 80 nM were identified by screening libraries containing 800 million synthetic peptides with 13 residue-long sequences using affinity selection-mass spectrometry.^118^ However, the developed peptide does not inhibit interaction of the RBD with ACE2.

Stapling of peptides—covalently joining residues close in a space in a specific conformation—can be efficiently applied to rigidify helical peptidomimetics that could bind RBD. Although initial attempts to staple ACE2 fragments were unsuccessful,^119^ further studies provided molecules with micromolar potency.^120−122^ Moreover, macrocyclic peptides were also discovered using epitope-directed selection in phage display.^123^

**Figure 14:**
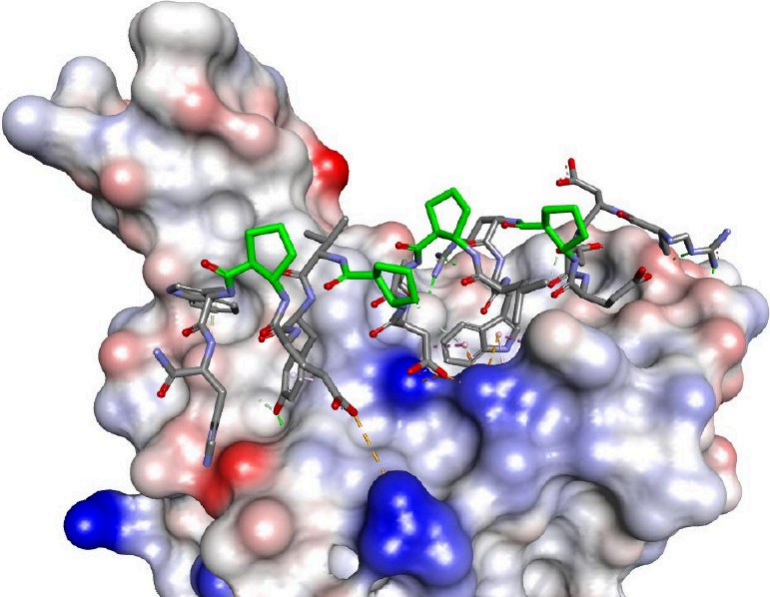
Modeled complex of peptide-RBD of the S protein. The inhibitory peptide is shown in stick representation colored according to atom type, and β-amino acid carbon atoms are colored in green. The RBD of the S protein is shown as a solvent-accessible surface that is colored according to interpolated charge (red–negative, blue–positive, gray–neutral). Interactions are shown as dashed lines with the same color scheme as shown in Figure 1.

On the basis of the sequence of the helical fragment of the LCB3 protein, conformationally constrained peptides were developed.^124^ Incorporation of a cyclopentane-based β-amino acid residue using an ααβαααβ sequence pattern resulted in peptides retaining a helical conformation in solution. After four rounds of optimization, the peptide showed binding affinity to the RBD of the S protein, *K*_d_ = 650 nM. Moreover, the HTRF assay allowed us to confirm the inhibition of the S protein–human ACE2 interaction by engineered peptides with an IC_50_ equal to 1.3 μM. Modeling studies revealed the importance of two Trp residues that hook the peptide in the RBD, which were accompanied by several charge-assisted hydrogen bonds (Figure 14).

Two peptides, HR1–1 and HR2–18, designed to inhibit the interaction between the HR1 and HR2 domains, thus blocking the formation of the six-helix bundle of the S protein, were identified as potential inhibitors, with EC_50_ values of 0.14 and 1.19 μM, respectively, as determined using the wild-type SARS-CoV assay.^125^ Additionally, two peptides derived from ACE2, containing charged amino acids (residues 22–44 and 22–57), possess SARS-CoV antiviral activity with IC_50_ values of 50 and 6 μM, respectively.^126^ However, another peptide comprising two ACE2 segments (residues 22–44 and 351–357) linked by glycine showed stronger activity in the inhibition of SARS-CoV infection, with an IC_50_ of approximately 0.1 μM.

The first pan-CoV fusion inhibitor named EK1 was developed on the basis of the HR2 sequence in the S protein of HCoV-OC439.^127^ This peptide was effective in inhibiting various HCoVs by forming a stable six-helix bundle with the HR1 domain, as confirmed by crystal structures. The EK1 peptide was further modified by the introduction of polyethylene glycol (PEG) and cholesterol, resulting in lipopeptide EK1C4 with significantly improved inhibitory activity against all HCoVs tested, including SARS-CoV-2.^128,129^ EK1C4 effectively inhibits SARS-CoV-2 replication with an EC_50_ value of 36.5 nM, which is 67-fold more potent than EK1 (EC_50_ = 2.47 μM) in the same assay. However, due to the existing side effects and risks involved with the use of PEGylated drugs,^130,131^ a highly stable dePEGylated lipopeptide, EKL1C, was developed with broad-spectrum anti-HCoV activity, a high genetic barrier for drug resistance, improved resistance to proteolytic enzymes, and higher thermostability.^132^ In addition, fusion inhibitor peptides targeting the spike S2 subunit were studied, revealing a long 36-mer peptide with potent inhibition of SARS-CoV-2 S-mediated cell–cell fusion at a 1 μM concentration.^133^ Another study showed that the combination of the fusion inhibitor EK1 and an antiviral lectin, griffithsin (GRFT),^134^ displays an excellent synergistic effect against SARS-CoV-2 pseudovirus infection, giving IC_50_ values of 49 nmol/L and 24 nmol/L, respectively.^135^ It is suggested that high inhibition potency is a result of GRFT and EK1 having different target sites, where GRFT inhibits SARS-CoV-2 infection by targeting the glycosylation sites in the S1 subunit, possibly the RBD, of the SARS-CoV-2 S protein.

The HR2 sequence-based lipopeptide fusion inhibitor IPB02 has high activity in inhibiting SARS-CoV-2, with an IC_50_ of 0.025 μM for the inhibition by IPB02 on SARS-CoV-2 S protein-mediated cell–cell fusion and IC_50_ values of 0.08 μM for the inhibition of SARS-CoV-2 pseudovirus infection. A structure–activity relationship study of IPB02 indicated the importance of the N- and C-terminal amino acids for binding and antiviral capacities.

An *in silico* study examined the potential of the HIV-1 fusion inhibitor Enfuvirtide for drug repurposing.^136^ The MD simulation study showed that interactions between Enfuvirtide and the most important residues of the HR2 domain of the SARS-CoV-2 S2 protein were remarkably stable, thus showing good potential to act as a fusion inhibitor of this virus.

### Small-Molecule Inhibitors

Modern discovery and development of safe drugs are most often a laborious and time-consuming process, often accompanied by a discouraging failure rate at the stage of clinical trials.^137^ To avoid these setbacks and accelerate the discovery of suitable therapeutics, research is directed to the repurposing of well-established drugs. The fast spread of COVID-19 infection has prompted the emergency use authorization of the antiviral drug remdesivir, even with its modest benefits.^138,139^

**Figure 15:**
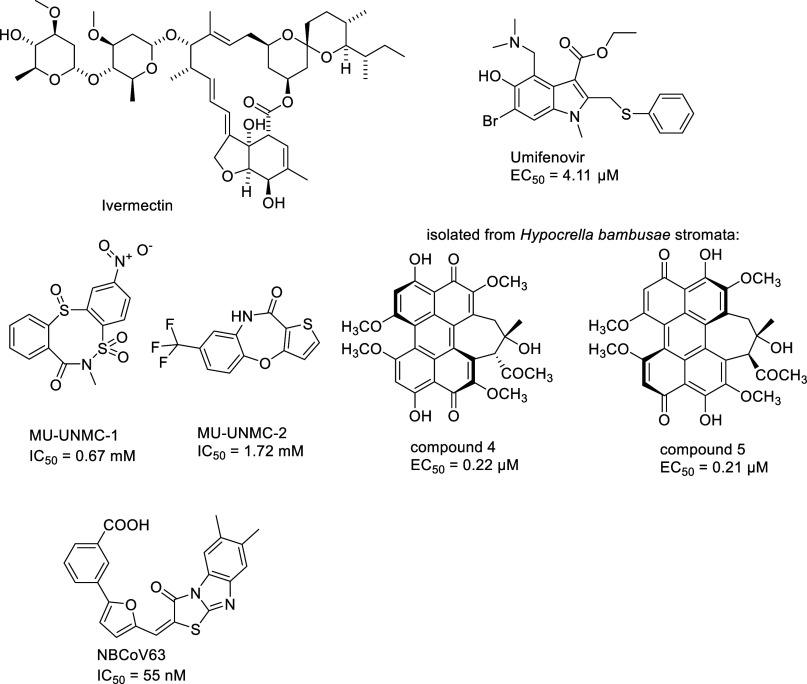
Structures of small molecules identified as S protein/ACE2 interaction inhibitors.

Ivermectin (Figure 15), a macrocyclic lactone derived from *Streptomyces avermitilis* and an FDA-approved antiparasitic, was found to inhibit the replication of SARS-CoV-2 *in vitro*,^140^ as successfully confirmed by MD simulation and docking calculations, which indicated that the R403, R405, Y449, L455, G496 and Y505 residues from ACE2 are the residues that interact the most with ligands.^141^ However, no clinical evidence was found showing that it is effective in COVID-19 treatment.^142^ Furthermore, an anti-influenza drug, umifenovir, was found to efficiently inhibit SARS-CoV-2 infection with an EC_50_ of 4.11 μM.^143^ MD simulation-based analyses demonstrated a high affinity binding of umifenovir at the RBD/ACE2 interface (Figure 15), inducing structural rigidity in the viral glycoprotein, thus limiting the conformational rearrangements needed for membrane fusion and virus entry.^144^

Numerous *in silico* and *in vitro* screening studies, considering both FDA-approved drugs and newly designed structures, were performed to identify highly bioactive molecules that could efficiently suppress COVID-19 infection.^145,146^ Natural products, such as alkaloids, could have good potential for treating different diseases with mild side effects. An investigation was conducted to evaluate the potential of alkaloids to interact with and alter the binding function of the SARS-CoV-2 S protein and block the receptor function of ACE2.^147^

In a computer-aided drug design approach, two potential inhibitors of the S protein RBD and ACE2 interaction, named MU-UNMC-1 and MU-UNMC-2 (Figure 15), were identified and validated.^148^ In SARS-CoV-2 infection assays, both compounds showed antiviral activity, with IC_50_ values of 0.67 and 1.72 mM, respectively. Moreover, MU-UNMC-2 has a synergistic effect when combined with remdesivir, implying the possibility of developing a combination therapy to treat COVID-19 patients.

Compounds (Figure 15) isolated from the stromata of *Hypocrella bambusae* were characterized as potential SARS-CoV-2 entry inhibitors, showing potent activity against live SARS-CoV-2 infection with EC_50_ values of 0.22 and 0.21 μM, respectively.^149^ Moreover, cell–cell fusion assays, surface plasmon resonance assays, and molecular docking studies revealed that these compounds could bind with the RBD of the SARS-CoV-2 S protein to prevent its interaction with the human ACE2 receptor.

Recently, several highly efficient small molecule pan-coronavirus inhibitors were discovered.^150^ Among them, the compound NBCoV63 (Figure 15) showed a high potency against SARS-CoV-2 (IC_50_ = 55 nM), SARS-CoV (IC_50_ = 59 nM), and MERS-CoV (IC_50_ = 75 nM) with excellent SI values and favorable *in vitro* ADME properties.

## Hepatitis C Virus Inhibitors

The hepatitis C virus causes an infection that can have life-long consequences, becoming chronic and eventually leading to liver cirrhosis and hepatocellular carcinoma.^151^ It is an enveloped ssRNA virus with seven recognized genotypes and high genetic diversity, making it a difficult target for vaccine and drug development.^151,152^ The current treatment employs FDA-approved direct-acting antivirals (DAAs) and their pangenotypic combinations. Nonetheless, this solution has two significant drawbacks: the high price of the treatment and the emergence of resistance-associated variants.^151−153^

An alternative approach is focused on the inhibition of the entry of viruses into hepatocytes. It is a complicated, multistep mechanism involving many host factors, such as the tetraspanin CD81, scavenger receptor class B member 1 (SR-BI), claudin-1 (CLDN1), and occludin.^154^ It requires two viral envelope glycoproteins, E1 and E2, which form a heterodimer on the surface of the virus. Although only E2 interacts directly with the entry factors, E1 assists it at different stages of the process; hence, it may be crucial to consider these two together as a complex.^155,156^

**Figure 16:**
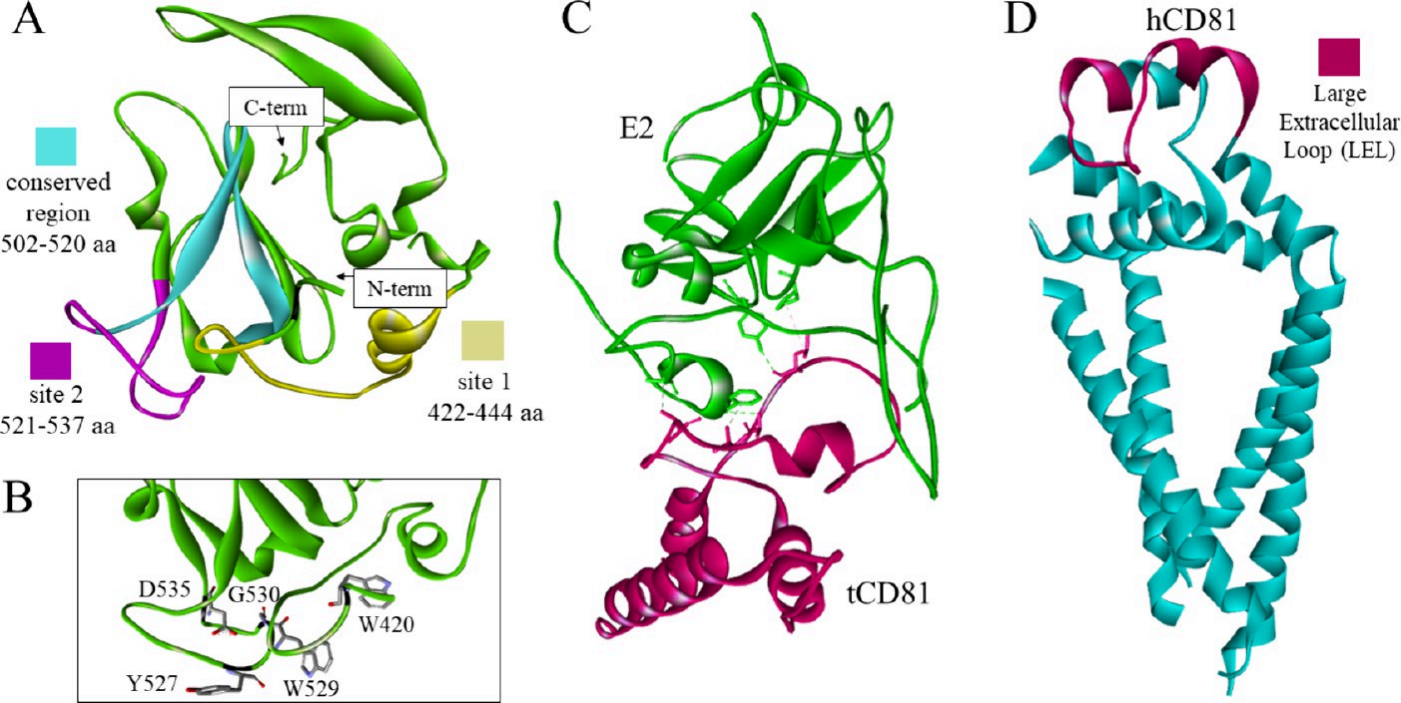
(A) The crystal structure of the truncated glycoprotein E2 shown in green in ribbon representation with fragments containing the predicted binding sites in yellow and magenta, as well as a conserved region in cyan (PDB id: 6MEH).^157^ (B) Amino acid residues crucial for the E2-CD81 interaction, presented as sticks colored according to atom type.^158^ (C) The interaction between tamarin CD81 (pink) and E2 (green) with the interacting residues carbon atoms’ colors matching the ribbon color of the same chain (PDB id: 7MWX).^159^ (D) The full structure of human tetraspanin CD81 in the closed conformation, including the transmembrane helices with a cholesterol binding pocket.^160^ The large extracellular loop is highlighted in pink (PDB id: 5TCX).^160^

The development of entry inhibitors is hampered by the lack of crystal structures of the complexes involved. The most well-studied host factor is CD81, a transmembrane protein from the tetraspanin family, which interacts directly with E2 glycoprotein. Unfortunately, CD81 is ubiquitously expressed in the human body and has multiple functions, raising doubts about the potential toxicity of its inhibitors. The problem is further complicated by the conformational flexibility of both CD81 and E2. Based on the changing position of the large external loop (Figure 16) on results from X-ray crystallography, CD81 is thought to adopt three conformations—open, closed, and intermediate.^161^ It is possible that E2 and CD81 undergo conformational changes upon binding.^161,162^ To date, there is no solved structure of the complex.^163^ Nonetheless, attempts to dock the interacting domains using molecular modeling methods have been made,^164^ and a structure of the complex with tamarin CD81 has been resolved (Figure 16C).^159^ It has been determined that the glycoprotein’s amino acid residues W420, Y527, W529, G530, and D535 are crucial for the CD81-E2 interaction (Figure 16B)^158^ and that the E2 502–520 aa segment is conserved and necessary for HCV infectivity.^165^ Additionally, molecular dynamics (MD) simulations predict that E2 binds to CD81 at site 1 (422–444 aa) at first, undergoes conformational changes, and then binds at site 2 (521–537 aa, Figure 16A).^164^

CD81 is not the only promising target for the treatment of HCV infection. SR-BI (also known as SRB1) is an HDL receptor participating in the bidirectional transport of cholesterol. Its involvement in cell entry can be 2-fold: direct interaction with the E2 glycoprotein and with lipoproteins associated with the virion.^166,167^

The tight junction proteins start playing a larger role in the late stages of entry. **CLDN1** is a tight junction protein that mediates paracellular permeability and is highly expressed in the liver. It forms a coreceptor with CD81 after the formation of the HCV-CD81 complex.^168^ The disruption of the CD81-CLDN1 interaction has become one of the targets for drug development. While a full structure of CLDN1 has not been solved, claudins are composed of two extracellular loops and four transmembrane helices.^169^ Mutations of amino acid residues 32 and 48 of CLDN1 extracellular loop 1 have revealed their importance for HCV infectivity.^170^

### Antibody-Based Inhibitors

The glycoprotein E2 is highly immunogenic; hence, triggering the natural body response has become the focal point of vaccine development. E2 has multiple regions with high sequence variability, facilitating virus escape from neutralization. Structural studies of the E2 glycoprotein point to a couple of neutralization epitopes with conserved residues, the identification of which has led to the development of broadly neutralizing antibodies, such as HC84.26.5D,^171^ AR3A,^172,173^ and AP33 (Table 2).^153,174^ While many of the developed antibodies are in late-stage development, there are still questions regarding their long-term usefulness. It is possible that further viral evolution and mutation of the conserved residues will lead to the emergence of new resistance-associated variants.^171^

**Table 2:** Antibodies Targeting HCV Entry, Their Epitopes, and the Reported IC_50_

name	target	epitope	IC_50_
AR3A^172,173^	E2	L427, N428, C429, N430, D431, T439, F442, Y443, W529, E531	0.5 nM < 0.002 μM
HC84.26.5D^171^		W437, F442, Y443, K446	0.0013–0.0082 μM^a^
AP33^153,174^		412–423 aa (QLINTNGSWHIN)	0.013–0.69 μM^a^
K21^175^	CD81	LEL	56 ± 11 ng/mL
JS81^175^		LEL	144 ± 43 ng/mL
mAb151^176^	SRBI	Not determined	0.5 nM
7A5^177^	CLDN1	M152 (second extracellular loop)	0.23 μg/mL

a Depending on the HCV variant.

**Figure 17:**
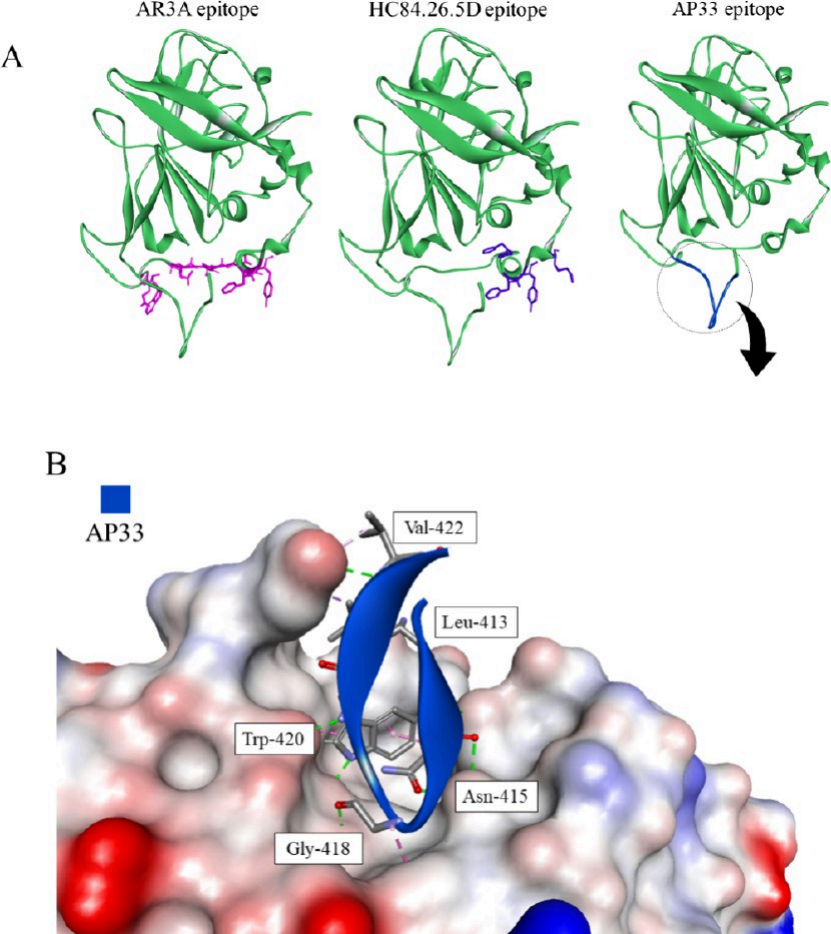
(A) The epitopes of the E2-targeting antibodies. The proteins are shown in cartoon representation with the epitope amino acid side chains displayed as sticks (PDB id: 6MEI).^157^ (B) The intermolecular interactions between AP33 and its epitope are shown in two ways: on the left, the epitope is represented as a blue ribbon with the interacting amino acid residues as sticks. On the right, the antibody is the solvent accessible surface colored according to interpolated charge (red–negative, blue–positive, gray–neutral), with the interacting residues of E2 displayed as blue sticks. Interactions are shown as dashed lines with the same color scheme shown in Figure 1 (PDB id: 4GAG).^153^

Host factors have also been targeted for vaccine development. Monoclonal antibodies against CD81,^175^ SR-BI,^176^ and CLDN1^177^ have been developed (Figure 17). This approach should prevent the appearance of resistance-associated variants. However, the initial function of the proteins could be impacted, so the binding site should be chosen carefully. Antibodies are also much better at preventing infections than at clearing an existing one, and their cost and instability may limit the availability of the treatment.

### Peptide-Based Inhibitors

Much research has been performed in the pursuit of peptide-based HCV entry inhibitors. The rational design of inhibitors is often based on the structure of the binding partner. The peptide 710–725 is based on the C-terminal sequence of E2 and has been determined to inhibit the CD81-E2 interaction, showing that this region of the glycoprotein also participates in binding to CD81 (Table 3).^178^ This methodology was also applied to create the CLDN1 N-terminus-derived peptide CL58, which prevents the formation of the CD81-CLDN1 complex.^179^ Furthermore, screening of a library of peptides made out of 15-amino acid fragments of E1E2 yielded the peptide E2–42 (E2 residues 548–562), which can interact with both glycoproteins.^180^

**Table 3:** Peptide-Based Inhibitors of HCV Entry, Their Sequences, and the Available Inhibition Data

target	name	sequence	efficiency of inhibition
CD81	710–725^178^	ASWAIKWEYVVLLFLL	∼60% relative luciferase activity at 51 μM^a^
	CD81-binding nonapeptide^182^	SPQYWTGA motif	NA
	CL58^179^	MANAGLQLLGFILAFLGW	IC_50_ = 2.1 ± 0.5 μM
E2	hCD81-like nonapeptide^184^	ATWVCGPCT	E2 inhibits binding of C9 clone to anti-hCD81 JS-81 with inhibition ratio of 67.5%
	SAHH-5^181^	PSGSN**X**SNIISN**X**FRED	EC_50_ = 17–39 μM^b^
	C18^183^	WPWHNHR	Relative level of HCV RNA ∼ 0.5 at 97 μM^c^
	E2–42^180^	QGSWFGCTWMNSTGF	% of infectivity at C = 5 nM: 50–60%

a Luciferase activity in Huh7.5 cells compared to the activity in PBS buffer.

b Value depending on the HCV subtype.

c In Huh7.5 cells infected with HCVcc.

The properties of peptide inhibitors can be further improved by adding stabilizing modifications. SAHH-5, a peptide based on the large extracellular loop of CD81, was stapled through a covalent bond between two amino acid side chains, ensuring proper folding and high stability against proteolysis.^181^

Another way to find active anti-HCV compounds is through phage display selection. Curiously, CD81-binding nonapeptides selected from a random peptide library did not share any sequence similarities with E2. The structural motif present in all of the CD81-binding peptides found in the study is speculated to be a mimotope of an E2 epitope.^182^ Similarly, a set of short peptides was found to bind to E2.^183^

### Small Molecule-Based Inhibitors

**Figure 18:**
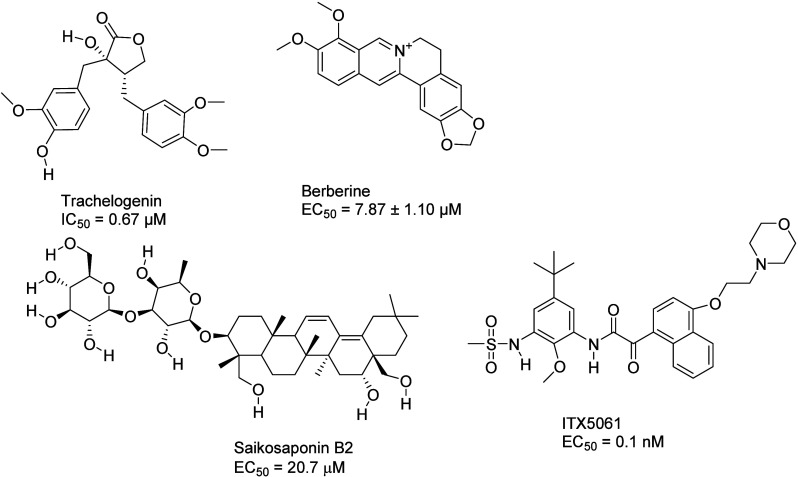
Small molecule inhibitors of HCV entry and available inhibition data.

The recent trend is to aim for naturally sourced inhibitors characterized by their affordability and availability even in disadvantaged communities. Such molecules have also been found to inhibit the protein–protein interactions occurring during HCV entry. Trachelogenin (Figure 18), a natural lignan compound obtained from *Caulis trachelospermi*, disrupts the E2-CD81 interaction by binding to the large extracellular loop of CD81.^185^ According to MD simulations, it interacts with residues T166, N184, K187, and E188.^185^

Saikoponin B2 (Figure 18) is a terpenoid that can be extracted from the roots of *Bupleurum kaoi.* It targets the E2 glycoprotein, effectively preventing its binding to CD81. Furthermore, its effect may be pangenetic.^186^ The plant alkaloid berberine (Figure 18) has a similar mode of action and blocks the entry of HCV into cells. Its binding site may be close to site 1, which is involved in the formation of the complex with CD81.^187^

ITX5061 (Figure 18), an SR-BI antagonist, has reached phase I clinical trials. The arylketoamide hampers the activity of SR-BI but also stops the binding of a soluble, truncated form of E2 to the receptor. Whether SR-BI binding with E2 directly occurs during the entry process is disputed.^188^

## Ebola Virus

**Figure 19:**
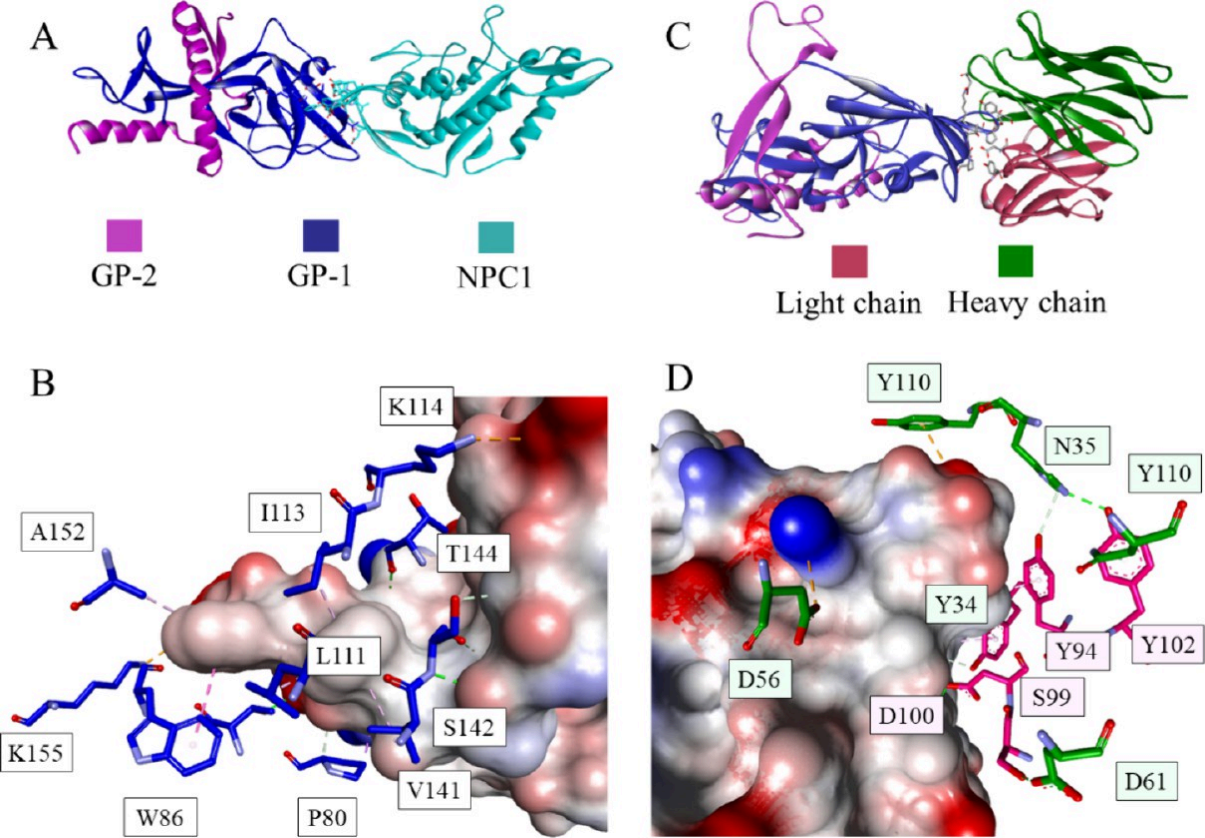
Glycoprotein GP in the complex with the Niemann-Pick C1 receptor (A) (PDB id: 5F1B)^195^ and closeup showing intermolecular interactions (B). The glycoprotein GP in the complex with the IT0227 antibody (C), with a closeup of the interactions shown in (D) (PDB id: 6S8D).^196^ The proteins are shown in ribbon representation colored by chain with magenta for GP-2, navy for GP-1, cyan for NPC1, red for the light chain of IT0227, and green for the heavy chain of IT01227. Interacting residues are shown as sticks colored by atom type, and the color of the carbon atoms matches that of the ribbon of the same chain. Interactions are shown as dashed lines with the same color scheme shown in Figure 1.

**Figure 20:**
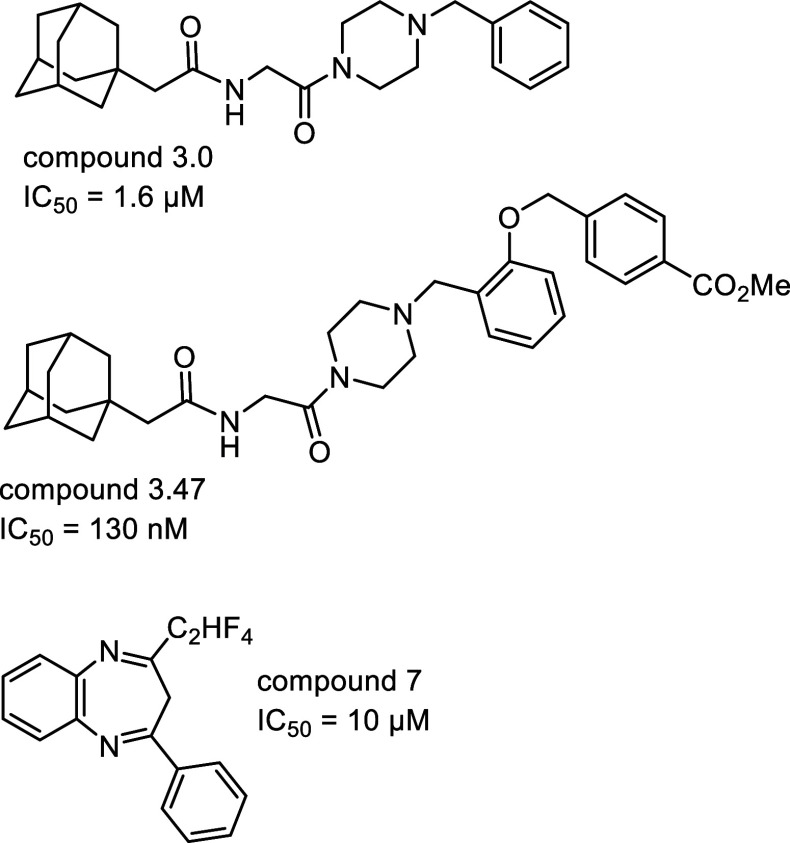
Small molecule inhibitors of PPI related to the Ebola virus.

The 2014–2016 outbreak brought much attention to the Ebola virus. The enveloped filovirus causes a severe hemorrhagic fever in humans. Currently, there are three FDA-approved vaccines against it; nonetheless, studies of entry inhibition have also progressed. The virus has one glycoprotein (GP) on its surface, which is cleaved into two separate parts postattachment.^189,190^ The first part, GP-1, is involved in receptor interactions and has a Niemann-Pick C1 receptor-binding domain (Figure 19A,B). GP-2 is crucial for the membrane fusion step. The antibodies isolated from vaccinated individuals, such as the 1T0227 antibody, have been proven to bind at the receptor binding domain of GP-1 (Figure 19C,D).^190^ Furthermore, the structure of the resolved NPC1-GP complex has been used to develop a set of macrocyclic peptide analogs of loop 2 of the host factor. The highest inhibitory activity was achieved with Pep3.3 (IC_50_ = 5.1 μM, Ac-cyclo(CEYFFWYC)-NH_2_).^191^ Two small molecule inhibitors of the interaction have been found through library screening; the compounds, benzylpiperazine adamantane diamide (compound 3.0, IC_50_ = 1.6 μM) and its derivative, compound 3.47 (IC_50_ = 130 nM), target NPC1 directly, but the exact mode of binding is unknown (Figure 20).^192^ Many studies have focused on inhibitors targeting a loop at the interface between GP1/GP2, blocking the fusion of the virus and the cell. Nonetheless, the conformational changes and the structural destabilization of GP can disrupt the interaction with NPC1 as well.^193^ For example, a benzodiazepine derivative (compound 7, IC_50_ = 10 μM, Figure 20) targets a hydrophobic pocket at the interface of this interaction. Whether it affects the glycoprotein or only prevents its binding to NPC1 is yet unclear.^194^

## Dengue Virus

Dengue fever, despite its annual 390 million human infections,^197^ continues to be classified as a neglected tropical disease.^198^ It predominantly affects low-income and developing regions of the world.^199^ The severe form of the disease can be life-threatening and can lead to dengue hemorrhagic fever or dengue shock syndrome. The risk is elevated for individuals who have already been infected by a different strain of the virus possibly due to antibody-dependent enhancement of infection (ADE). The existence of four distinct serotypes with sequence similarity of 60–70% is the main challenge for vaccine and treatment development.^199^ Currently, two vaccines have been authorized, but none have been authorized by the FDA. The European Medicines Agency approved the Qdenga tetravalent attenuated vaccine in 2022.^200^ Another vaccine, Dengvaxia, has been available since 2016.^201^ The latter is recommended only for previously infected individuals and populations with high infection rates.

**Figure 21:**
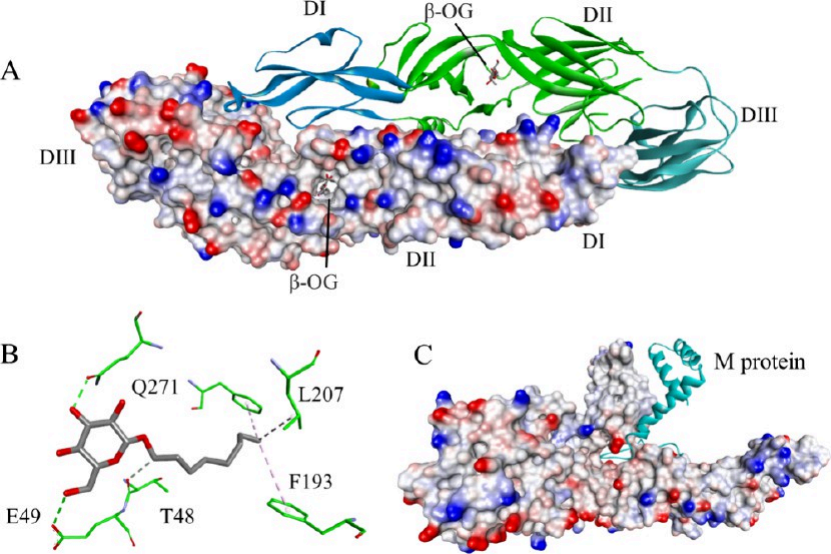
(A) The crystal structure of a DENV2 envelope glycoprotein homodimer (PDB id: 1OKE).^202^ One chain is shown in the ribbon representation with blue marking the DI, green – DII, and cyan – DIII. The other chain is shown as a solvent-accessible surface colored according to the interpolated charge (red–negative, blue–positive, gray–neutral). The binding sites of *n*-octyl-β-d-glucoside are indicated with an arrow (“β-OG”). (B) Interactions within the *n*-octyl-β-d-glucoside-binding pocket; the ligand and the amino acid residues are shown in stick representation, with colors corresponding to atom type. The protein’s carbon atom color corresponds to the location of the binding site (green - DII). Interactions are shown as dashed lines with the same color scheme shown in Figure 1. (C) The E protein–M protein complex. Both envelope protein chains are depicted in the solvent accessible surface representation, while the M protein is shown as a cyan ribbon (PDB id: 3J27).^206^

Dengue virus (DENV) is an enveloped *flavivirus* that encodes three structural proteins: capsid (C), premembrane (prM), and envelope (E). The spikes on the virion particles are composed of three prM:E heterodimers. The envelope glycoprotein has three domains. Domain III (DIII) contains immunogenic epitopes and receptor-binding motifs, while the hydrophobic membrane fusion loop is located in domain II (DII) (Figure 21A).^202^ The entry route of DENV seems to be serotype dependent. Multiple host receptors have been identified for their role in the process, including DC-SIGN, CLEC5A, mannose receptor, CD14, heat-shock proteins 70 and 90, ER chaperonin GRP78, and prohibitin.^203^

**Figure 22:**
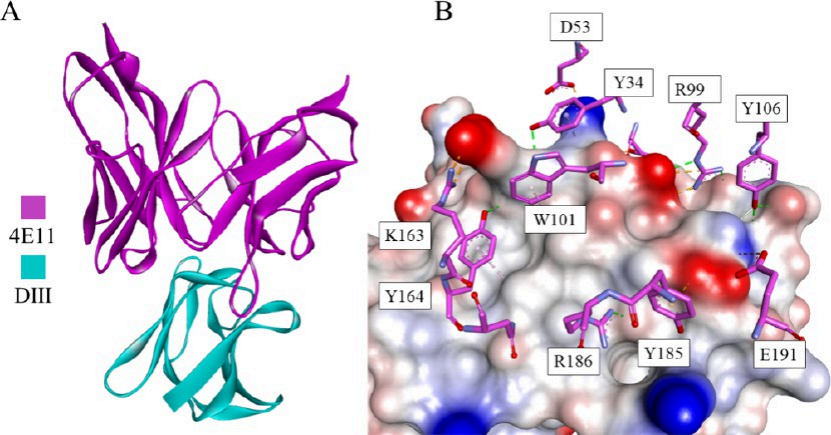
(A) The 4E11 bNAb-DIII complex (DENV4) is shown in the ribbon representation, where 4E11 is colored magenta, while DIII is colored cyan. (B) A closeup showing the intermolecular interactions. DIII is shown as a solvent-accessible surface colored according to interpolated charge (red–negative, blue–positive, gray–neutral). The interacting residues are displayed as sticks colored by atom type, and the colors of the carbon atoms match that of the ribbon of the same chain. Interactions are shown as dashed lines with the same color scheme shown in Figure 1 (PDB ID: 3UYP).^207^

It is crucial to immunize the patient against all DENV strains to prevent the onset of severe disease. The murine broadly neutralizing antibody 4E11 (Figure 22) exhibits a binding affinity to the DIII region of four DENV serotypes (DENV1 to DENV4), although with comparatively weaker binding to DENV4.^204^ This antibody recognizes a conserved fragment within DIII, encompassing residues K310, L/I387, L389, and W391.^204^ It was further re-engineered to enhance its binding capabilities, resulting in the 4E5A antibody. Finally, further optimization of 4E5A yielded Ab513 with EC_50_ values below 200 ng/mL for all serotypes.^205^

Due to the uncertainty in the role of the suspected host factors as well as the serotype dependency of the entry mechanism, most work on DENV entry inhibition has been focused on the structural proteins of the virus.

**Figure 23:**
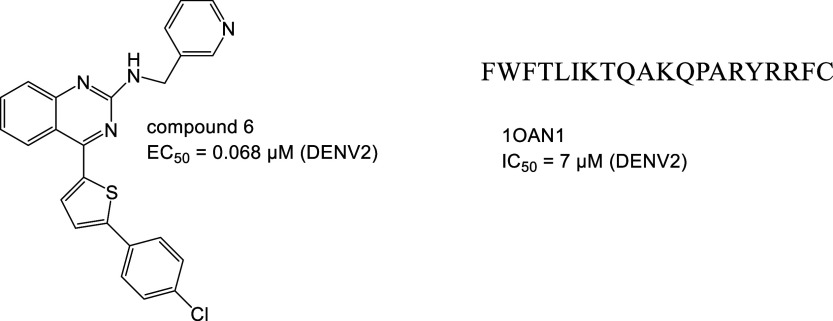
Inhibitors of PPI involved in dengue infection.

During maturation in the trans-Golgi network, the prM protein is cleaved by a protease into two parts: the M protein and the pr peptide. This triggers a conformational change in E and primes the particle for membrane fusion.^203,202^ The crystal structure of the envelope protein revealed an *n*-octyl-β-d-glucoside (β-OG)-binding hydrophobic pocket that is essential for the structural transition (Figure 21B).^202^ Targeting this pocket has become one of the main strategies for entry inhibition. The EF dipeptide targets this site, resulting in an IC_50_ of 96.8 μM (DENV2),^208^ as does Compound 6 (Figure 23) with an EC_50_ of 0.068 μM (DENV2).^209^ Another site is targeted by 1OAN1, a *de novo* peptide binding to the 41–60 amino acid fragment of the envelope protein. *In silico* optimization of the sequence resulted in an IC_50_ of 7 μM (DENV2).^210^

MLH40, a peptide created from the conserved ectodomain of the M protein, interferes with the M-E interaction, changing the viral interface and hence blocking entry at the attachment stage. The value of IC_50_ depended on the serotype, with results between 24 and 31 μM.^211^

## Chikungunya Virus

Chikungunya virus (CHIKV) is an enveloped *alphavirus* originating from Africa. The disease is characterized by high fever and joint and skeletal muscle pain. While it is not deadly to otherwise healthy individuals, it can become chronic and significantly lower the quality of life.^212^ Currently, there is no vaccine or specific treatment for CHIKV; nonetheless, some potential vaccines are in clinical trials and on their way to approval, such as live-attenuated VLA1553.^213,214^

**Figure 24:**
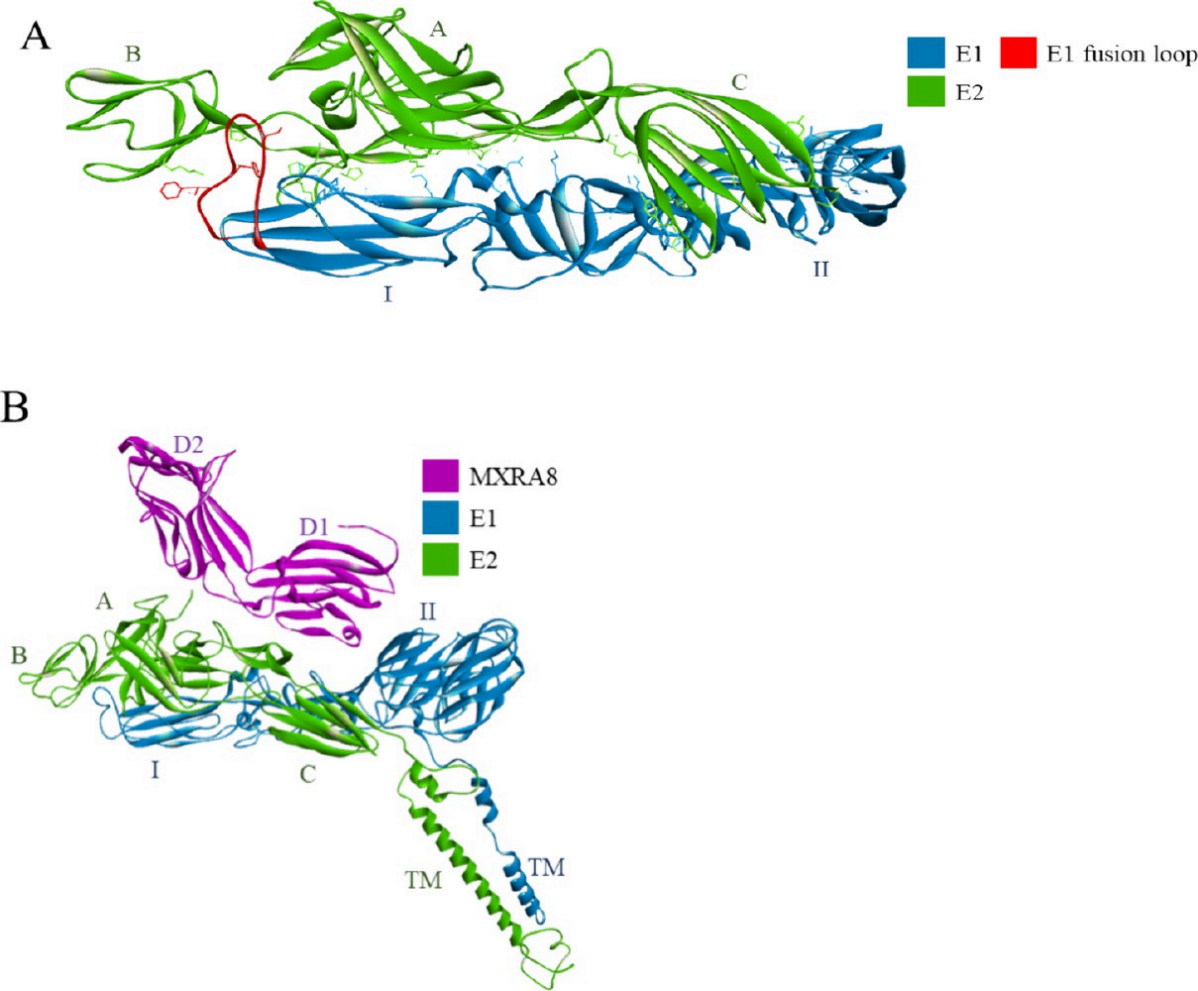
(A) The E1E2 complex of CHIKV. The structure is represented as ribbons, with green indicating the E2 glycoprotein, blue indicating the E1 glycoprotein, and red indicating the fusion loop. The amino acid residues involved in the intramolecular interactions between E1 and E2 are shown as sticks. (B) The MXRA8 receptor (magenta ribbon) binds to the E1E2 complex. The transmembrane part of E1E2 is included (“TM”) (PDB id: 6JO8).^223^

CHIKV enters host cells through pH-dependent receptor-mediated endocytosis. The viral glycoproteins E1 and E2 form heterodimeric spikes on their surfaces, with the former participating in membrane fusion and the latter interacting directly with host factors. The acidic pH of endosomes is speculated to cause a conformational change in the E1E2 complex (Figure 24), resulting in exposure of the hydrophobic fusion loop of E1, which acts as an anchor in the cell membrane.^212^

**Figure 25:**
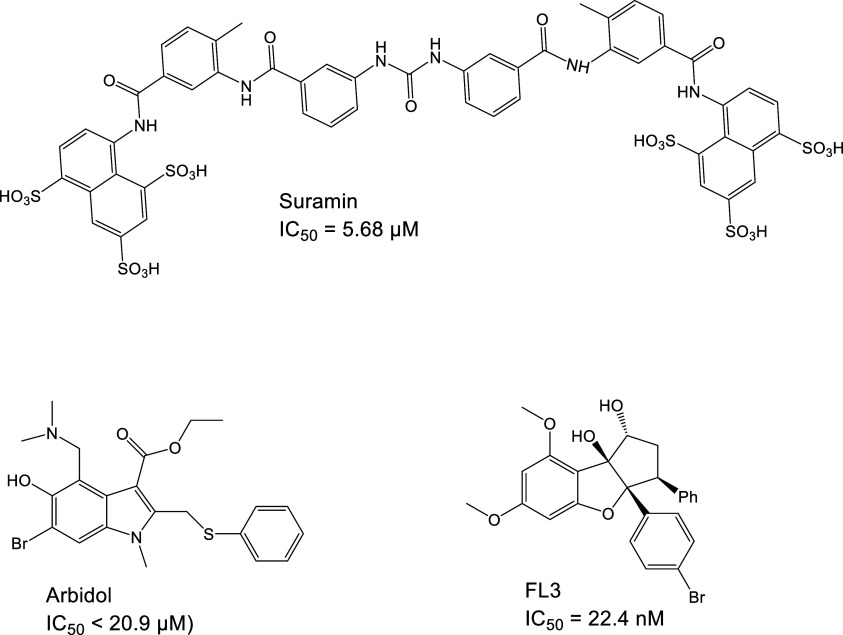
Small molecule inhibitors of PPI related to Chikungunya virus infection.

E2 is composed of three domains, one of which—domain A—is responsible for receptor binding. The C9 (IC_50_= 51 ng/mL) and IM-CKV063 (7.4 ng/mL) neutralizing antibodies interact with the domain of one E2 glycoprotein and domains A and B of a neighboring E2 in the spike.^215^ It has also been reported that one residue, E2 W64, is especially important and may be an attractive target for drug and vaccine design.^215^ E2 has also been targeted by small molecule inhibitors, such as suramin (IC_50_ = 5.68 μM)^212^ and Arbidol (IC_50_ < 20.9 μM) (Figure 25).^216^ It has been found that the N5R H18Q mutation of E2 results in a suramin-resistant variant, while the G407R substitution causes Arbidol resistance.^212,216^ Furthermore, the N-terminal loop and domain A of E2 have been identified *in silico* as the most likely suramin binding site.^212^

Phospholipase A2, a snake venom protein, is a strong CHIKV entry inhibitor with an EC_50_ of 44.6 nM; according to MD simulations, it may bind to E1, forming approximately 30 hydrophobic interactions.^217^ Three amphiphilic peptides, GA-Hecate (gallic acid-FALALKALKKALKKLKKALKKAL-CONH_2_) and its analogs, PSSct1910 and PSSct1905, have been reported to interfere with virus attachment and internalization into cells. Among these peptides, PSSct1910 showed the most promising results, with an EC_50_ of 1.1 μM in BHK-21 cells and 0.2 μM in Huh-7 cells.^218^

To date, several possible host receptors have been proposed, including the MXRA8 receptor (Figure 24B), prohibitin-1, TIM1, DC-SIGN, and the basigin CD147.^219,220^ The E2/prohibitin-1 interaction was inhibited by the small molecule drug flavagline FL3, which binds prohibitin-1 with an IC_50_ of 22.4 nM (Figure 25).^221^ Despite the recognition of MXRA8 as a host factor in CHIKV entry, its decrease in concentration or absence did not fully stop the infection, suggesting the existence of an MXRA8-independent entry pathway.^222^

## Summary and Outlook

Usually, in the case of newly emerging viral diseases, enzyme inhibitors are considered as a first choice due to their high efficiency combined with well-developed methodologies of their discovery. However, due to the high potential of viruses to change, exploration of various molecular targets is necessary. In particular, inhibitors of protein–protein interactions can be very valuable in the treatment of viral infections. In the majority of cases, interactions between the virus and human proteins are targeted, which affects the entry of the virus into the host cell. To efficiently create PPI inhibitors in these cases, detailed knowledge of the virus entry process is needed, and several proteins of both interacting partners are often involved. Analogous methodologies are applied to develop protein–protein inhibitors against any studied viruses, although the progress of projects is constrained by the availability of structural data and a comprehensive understanding of the viral entry mechanism. Furthermore, the lack of funding for research on rarely spreading diseases and/or in less developed countries poses an additional challenge.

Although the methodologies of development of viral PPI inhibitors are based on the same principles as those in other cases, some specific points can be indicated. The major difficulty of treating viral infections is related to fast mutations of viruses that can lead to changes in the structure of molecular targets and, subsequently, weakening of drug-target interactions. This problem can be addressed on two different levels, namely, molecule design and therapy development. First, drugs can be designed to bind regions of viral proteins that are less prone to mutations or target human proteins that interact with viral ones. Importantly, such options are not available in the case of enzyme inhibitor design. Second, antiviral therapy can be constructed in a way that drugs targeting various proteins are used at the same time. In this case, PPI and enzyme inhibitors can work cooperatively.

Usually, developing specific antibodies toward selected PPIs is the quickest option for obtaining highly active molecules, due to the availability of effective experimental methods. However, the high costs of antibody-based therapies and other disadvantages of antibodies are problematic in this case. Most often, peptides and peptidomimetics are the second choice. Recently, experimental and computational techniques have been significantly improved, and the effective development of compounds from this class is possible. Their medium size allows them to create compounds with numerous interaction sites with the protein target; thus, they can show high potency. On the other hand, the usual problems encountered in the medical application of peptides, e.g., poor proteolytic stability, can be feasibly solved by a wide range of known modifications leading to peptidomimetics. Finally, small molecules can also be applied as PPI inhibitors; however, their development is usually slower and requires more effort than that of the two classes discussed above. This is related to the necessity of finding a strong binder for large protein surfaces, which subsequently leads to extended compounds that are synthetically demanding.

Numerous successful stories presented in this Perspective confirm the high potential of PPI inhibitors in treating viral diseases. Both marketed drugs and drug candidates at various stages of clinical trials are known and constitute therapies that can be effectively applied alternatively or in combination with other approaches, particularly enzyme inhibitors. Advancements in methodologies of discovery and better possibilities to avoid virus resistance further enhance potential antiviral PPI inhibitors. Therefore, we can expect an increasing number of drugs derived from this field.

## Abbreviations Used

ACE2: angiotensyn converting enzyme 2
AIDS: acquired immunodeficiency syndrome
CCR5: C–C chemokine receptor type 5
CHR: C-terminal heptad repeat regions
CHIKV: Chikungunya virus
CXCR4: C-X-C chemokine receptor type 4
DAA: direct-acting antivirals
DENV: Dengue virus
FDA: Food and Drug Administration
HIV: human immunodeficiency virus
NHR: N-terminal heptad repeat regions
NTD: N-terminal domain
RBD: receptor binding domain
SARS: severe acute respiratory syndrome
PPI: protein–protein interaction
